# Proteomic profile of human sinoatrial and atrioventricular nodes in comparison to working myocardium

**DOI:** 10.1038/s41598-025-89255-y

**Published:** 2025-02-28

**Authors:** Agata Krawczyk-Ożóg, Aneta Stachowicz, Grzegorz Szoniec, Jakub Batko, Kamila Stachyra, Filip Bolechała, Marcin Strona, Paweł P. Wołkow, Zeyuan Yin, Halina Dobrzynski, Mateusz K. Hołda

**Affiliations:** 1 HEART - Heart Embryology and Anatomy Research Team, Department of Anatomy, Jagiellonian University Medical College, 12 Kopernika Street, Kraków, 31-034 Poland; 2 Department of Cardiology and Cardiovascular Interventions, University Hospital, Krakow, Poland; 3 Department of Pharmacology, Faculty of Medicine, Jagiellonian University Medical College, Krakow, Poland; 4 Department of Forensic Medicine, Jagiellonian University Medical College, Krakow, Poland; 5 Center for Medical Genomics OMICRON, Faculty of Medicine, Jagiellonian University Medical College, Krakow, Poland; 6 Division of Cardiovascular Sciences, The University of Manchester, Manchester, UK; 7 Division of Laboratory Diagnostics and Clinical Epigenetics, Faculty of Medicine, Institute of Medical Sciences, University of Rzeszów Medical College, Rzeszów, Poland

**Keywords:** Atrioventricular node, Proteomics, Right atrium, Right ventricle, Sinoatrial node, Cardiology, Proteomics

## Abstract

The proteomic profile of the human cardiac conduction system: the sinoatrial node (SAN) and atrioventricular node (AVN), remains poorly understood. The aim of the current study is to identify proteomic characteristic of the human SAN and AVN in the comparison to working myocardium of the right atrium (RAM) and right ventricle (RVM). The proteomic analysis was performed on 10 autopsied human heart specimens collected from healthy adults. During the data-independent acquisition proteomics analysis 2752 different proteins were identified in all sample sets. In both nodal tissues (compared to working myocardium), the following pathways were upregulated: regulation of Insulin-like Growth Factor transport and uptake by Insulin-like Growth Factor Binding Proteins, post-translational protein phosphorylation, glutathione metabolism, metabolism of carbohydrates, glycolysis and gluconeogenesis. Other common for nodal tissue pathways were these related to immune system and related to extracellular matrix. The pathways related to cardiac muscle contraction were more abundant in RAM and RVM samples. The current study presents extensive comparative analysis of protein abundance in the human SAN and AVN. Few key differences may be found in the nodal proteome in comparison to working cardiomyocytes, including involvement of immune system and upregulated pathways related to extracellular matrix. The SAN exhibits enrichment in the PPAR signaling and pentose phosphate pathways, as well as prostaglandin synthesis and regulatory proteins, compared to the AVN.

**Supplementary Information:**

The online version contains supplementary material available at 10.1038/s41598-025-89255-y.

## Introduction

The cardiac conduction system is composed of specialized cardiac myocytes with a unique embryological origin, as well as distinct anatomical, molecular, and functional properties that enable them to function collectively as the heart’s electrical system. It comprises several main components, such as the sinoatrial node (SAN), the atrioventricular conduction axis, including the atrioventricular node (AVN), its right and left bundle branches, and the terminal network of Purkinje fibers^1–3^ The human SAN, being the dominant pacemaker, is a ‘banana-shaped’ structure located subepicardially at the superior cavo-atrial junction of the right atrium (RA), with a tail extending along the terminal crest^4,5^. The AVN is located subendocardially in the apex of the Koch’s triangle, close to the central fibrous body of the heart^6^. Both SAN and AVN are not visible macroscopically, but may be exposed using histological techniques^7–10^. Recent years have also brought new experiential modalities such as micro-computer contrast-enhanced tomography that allows three-dimensional imaging of the individual components of cardiac conduction system^11,12^.

In a normal sinus rhythm, an impulse originates in the SAN, travels through atria and undergoes delay while passing through the AVN. Subsequently, it traverses the bundle of His, the left and right bundle branches, and ultimately enters the Purkinje fibers to stimulate ventricular contraction^13^. Any deviation from this conduction pathway leads to arrhythmia^14^ Abnormalities of cardiac rhythm are common diseases, affecting over 2% of adults^15^ Incidence of cardiac rhythm abnormalities occur at a yearly rate of 0.5%, a rate comparable to incidents of stroke, myocardial infarction, and heart failure^15^. Despite the development of several successful pharmacological and invasive treatment options for different forms of arrythmias, coping with some forms of arrhythmia is still suboptimal and sometimes characterised by unsatisfactory clinical results^16^.

There are many gaps in our knowledge on the nature, metabolism, or even the origin of particular elements of the electrical conduction system of the heart^17^. Especially, the proteomic profile of the human heart, and in particular its individual components, such as the SAN and AVN, remains poorly understood, with only few studies focusing on this topic^18–21^ However, a comprehensive understanding of the proteomic composition of various tissues is crucial for understanding the correlation between molecular phenotypes of cells and the clinical characteristics of organs^22^. The recognition of the proteomic characteristics of the electrical conduction cardiac system may give further information on the complex nature of the SAN and AVN^23–25^ The identification of protein markers specific to the SAN and AVN, akin to their signature or fingerprint, may allow for proteomic-based mapping of the cardiac conduction system^26^. Finally, understanding the proteomic profile of the SAN and AVN is of great interest as it holds the potential to facilitate the development of more effective diagnostic and treatment methods for arrhythmia^26,27^. Therefore, the aim of the current study was to characterize the proteomic profiles of the human SAN and AVN in comparison to the myocardium of the right atrium (RAM) and right ventricle (RVM), as well as to explore differences between the nodes, in a population of young healthy adults using state-of-the-art proteomics methods.

## Methods

### Sample collection

A total of 10 human heart specimens from healthy adults (30.0% females) with a mean age of 33.3 ± 14.3 years were collected during routine forensic medicine autopsies (12–16 h after death). The average body mass index (BMI) was 25.1 ± 3.6 kg/m^2^, and mean body surface area was 1.8 ± 0.2 m^2^. The main cause of death were traffic accidents, suicide and homicide. Exclusion criteria determined based on the patient’s medical history and autopsy examination included: severe anatomical defects, heart surgeries or heart grafts, evident severe macroscopic pathologies of the heart or vascular system found during autopsy (e.g. aneurysms, storage diseases), heart trauma, and previous history of any arrhythmias.

**Fig. 1:**
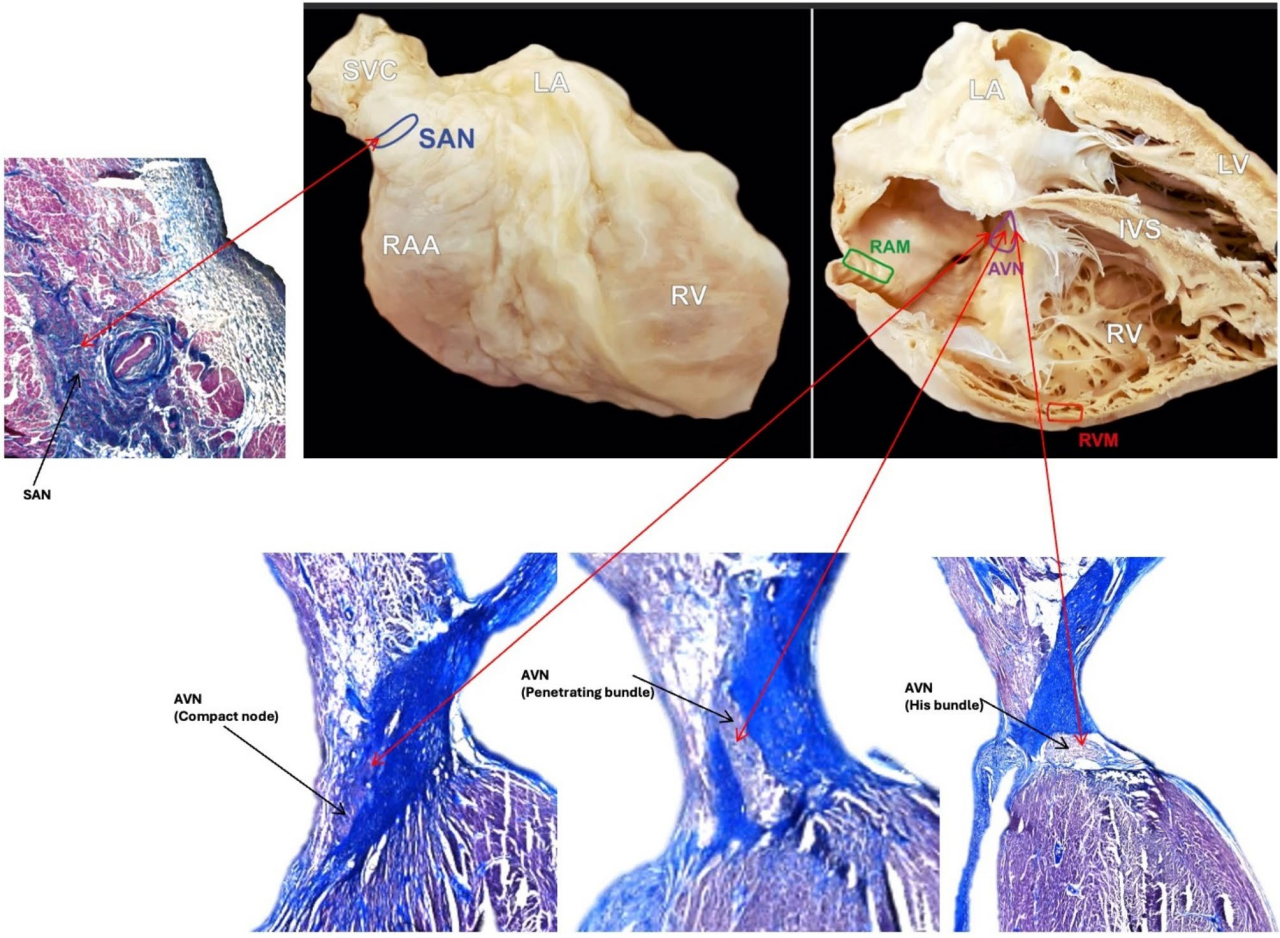
Photographs of cadaveric heart specimen showing four regions from where the samples were obtained: sinoatrial node (SAN), the atrioventricular node (AVN), working myocardium of the right atrium (RAM) and working myocardium of the right ventricle (RVM). Histological images (Masson’s trichrome staining) showing sections through the SAN and AVN (compact node, penetrating bundle and His bundle).

After dissection hearts were immediately perfused with mixture of saline solution with heparin and streptokinase to completely remove blood from the organ. With the use of microsurgical techniques and under the control of the operational microscope^28^ the following samples were collected (Fig. 1):

1. the SAN, located at the junction of the superior vena cava and the right atrium. After removal of the epicardium from this area and localizing the sinoatrial node artery, a block of the tissue (size 5 × 10 mm) was micro-dissected without the endocardium and epicardium^29^.
2. the AVN, located in the apex of the Koch’s triangle^6^. The endocardium was removed from this area and a block of the tissue (size 7 × 10 mm) was micro-dissected without the endocardium and epicardium^29^.
3. working myocardium of the right atrium (RAM), an atrial muscle taken from the right atrium (pectinate muscles), without endocardium and epicardium (size 10 × 10 mm).
4. working myocardium of the right ventricle (RVM), a working cardiomyocyte sample, taken from the right ventricle-free wall, without endocardium and epicardium (size 10 × 10 mm).

After dissection all samples were immediately deep frozen and stored at the − 80ºC temperature until further analyses.

## Histological examination

Thin central regions of SAN and AVN were dissected for histological analysis. Tissue blocks were embedded in paraffin before sectioning. Sections, 5 micrometers thick, were cut from each block and mounted on super adhesive glass (Super Frost Plus white Adhesion slides, Epredia). The Hematoxylin and Eosin staining was performed on serial sections (H&E Staining Kit, Abcam) and concurrently with the Masson Trichrome Staining Kit (Trichrome Stain [Masson] Kit, HT-15, Sigma-Aldrich) for collagen and muscle cells visualization, according to standard protocol. Stained slides were scanned with SLIDEVIEW VS200 (Olympus) and the images were retrieving using OlyVIA v.3.4.1 software (Olympus).

## Liquid chromatography-tandem mass spectrometry (LC‒MS/MS) analysis

Human heart samples were homogenized using a Tissue Lyser LT (Qiagen, Hilden, Germany) and lysed in a buffer containing 0.1 M Tris-HCl, pH 7.6, 2% sodium dodecyl sulfate, and 50 mM dithiothreitol (Sigma Aldrich, St. Louis, MO, USA) at 96 °C for 10 min. The protein concentration was determined by a Pierce 660 nm Protein Assay Kit (Thermo Scientific, Waltham, MA, USA). Seventy micrograms of protein were digested overnight using the filter-aided sample preparation (FASP) method^30^ with Trypsin/Lys-C mix (Promega, Madison, WI, USA) (enzyme-to-protein ratio 1:35) as the digestion enzymes. The amount of peptides after digestion was measured using a tryptophan fluorescence based WF-assay^31^. Next, the samples were purified with C18 Ultra-Micro Spin Columns (Harvard Apparatus, Holliston, MA, USA). For a spectral library preparation, equal amounts of peptides from all samples were subjected to a high-pH fractionation protocol on C18 Micro Spin Columns (Harvard Apparatus, Holliston, MA). Fractionation was carried out in 50 mM ammonium formate buffer (pH 10) with 12 consecutive elution steps with 5, 10, 12.5, 15, 17.5, 20, 22.5, 25, 27.5, 30, 35 and 50% acetonitrile in 50 mM ammonium formate buffer (pH 10). All samples and library fractions were dissolved in 0.1% formic acid at a concentration of 0.5 µg/µl and spiked with the iRT peptides (Biognosys, Schlieren, Switzerland).

One microgram of peptide was injected into a PepMap100 RP C18 75 μm i.d. × 25 cm column (Thermo Scientific, Waltham, MA, USA) via a PepMap100 RP C18 75 μm i.d. × 2 cm trap column (Thermo Scientific, Waltham, MA, USA) and separated using a 1–40% B phase linear gradient (A phase − 2% ACN and 0.1% FA; B phase − 80% ACN and 0.1% FA) with a flow rate of 300 nL/min on an UltiMate 3000 HPLC system (Thermo Scientific, Waltham, MA, USA) coupled to a TripleTOF 6600+ (Sciex, Framingham, MA, USA) mass spectrometer. The nanoelectrospray ion source (Optiflow, Sciex, Framingham, MA, USA) parameters were as follows: ion spray voltage: 3.2 kV; interface heater temperature (IHT): 200 °C; ion source gas 1 (GS1): 10; and curtain gas (CUR): 25. For data-dependent acquisition (DDA), spectra were collected for 135 min in full scan mode (350–1400 Da), followed by one hundred CID MS/MS scans of one hundred of the most intense precursor ions from the preceding survey full scan exceeding 100 cps intensity under dynamic exclusion criteria. For data-independent acquisition (DIA), spectra were collected for 100 min in full scan mode (400–1250 Da), followed by one hundred SWATH MS/MS scans using a variable precursor isolation window approach, with m/z windows ranging from 6 to 90 Da.

DDA MS data were searched against the human UniProt database and MaxQuant Contaminants list using the Pulsar search engine in Spectronaut software (Biognosys, Schlieren, Switzerland)^32^ with the following parameters: ± 40 ppm mass tolerance on MS1 and MS2 levels, mutated decoy generation method, Trypsin/Lys-C enzyme specificity, 1% protein and PSM false discovery rate (FDR). The library was generated using 3–6 fragment ions per precursor. The generated library was used to analyze DIA MS data in Spectronaut software. MS data were normalized and filtered by 1% FDR at the peptide and protein levels. Quantitation was performed at the MS2 level for peptide precursors that passed Q-value threshold in at least one of the samples. Missing values were imputed based on a random sampling from a distribution of low abundant signals taken across the entire experiment (global imputation). Statistical analysis of differential protein abundance was performed at both the MS1 and MS2 levels^33^ using unpaired t tests with multiple testing correction after Storey^34^. Individual heatmaps for each comparison were performed using RStudio.

## Enrichment analysis

Gene Set Enrichment Analysis (GSEA) identifies functional enrichment by comparing genes with predefined gene sets. We used the ClusterProfiler package for R^35^ to analyze enriched pathways based on the ReactomePA database^36^. We set adjusted p-values < 0.05 to filter nonimportant entries and plotted the top ten enriched ones.

## Constructing protein‒protein interaction networks

Functional grouping and pathway analysis were performed using PINE (Protein Interaction Network Extractor) software^37^ with the STRING and GeneMANIA databases using a score confidence of 0.4 and a ClueGO p value cutoff < 0.05. The mass spectrometry data have been deposited to the ProteomeXchange Consortium via the PRIDE partner repository^38^ with the dataset identifier PXD051011.

### Western blot analysis

Human heart samples were lysed in 2% SDS, 50 mM DTT in 0.1 M Tris-HCl (pH 7.6) as described in proteomics section. Tissue lysates containing equal amounts of total protein were mixed with 4x Laemmli Sample Buffer (Bio-Rad, Hercules, CA, USA) and incubated at 96 °C for 5 min. Protein samples (10 µg of protein per well) were separated on SDS-polyacrylamide gels (12% or 15%) using a Laemmli buffer system and then semidry transferred to nitrocellulose membranes by a Trans-Blot Turbo Transfer System (Bio-Rad, Hercules, CA, USA). The membranes were blocked with 5% nonfat dry milk in PBS at room temperature for 1 h and incubated overnight at 4 °C with specific anti-microfibril-associated glycoprotein 4 (Proteintech, Rosemont, IL, USA) (1:1000), anti-visinin-like protein 1 (Proteintech, Rosemont, IL, USA) (1:5000), anti-glycerol-3-phosphate dehydrogenase (Proteintech, Rosemont, IL, USA) (1:1000), anti-dynactin subunit 3 (BT LAB, Shanghai, China) (1:1000) and anti-glyceraldehyde-3-phosphate dehydrogenase (MyBioSource, San Diego, CA, USA) (1:2000) primary antibodies. Incubation with HRP-conjugated secondary antibodies (GE Healthcare, Chicago, IL, USA) was performed at room temperature for 1 h (dilution 1:10 000). Protein bands were developed by applying Clarity™ Western ECL Substrate (Bio-Rad, Hercules, CA, USA) for 5 min. Precision Plus Protein Kaleidoscope Standards (Bio-Rad, Hercules, CA, USA) were used for molecular weight determinations. Protein bands were visualized and imaged by using an ImageQuant LAS 500 scanner (GE Healthcare, Chicago, IL, USA).

### Statistical analysis

Data are expressed as the mean ± SEM. The equality of variance and normality of the data were checked by the Brown-Forsythe test and Shapiro-Wilk test, respectively. Based on the results, statistical analysis was performed using ordinary one-way ANOVA with correction for multiple comparisons by controlling the False Discovery Rate (two-stage linear step-up procedure of Benjamini, Krieger and Yekutieli) (Graphpad Prism 9.3.1, San Diego, CA, USA). Values of *p* < 0.05 (or q-values for proteomics experiments) were considered statistically significant.

## Results

**Fig. 2:**
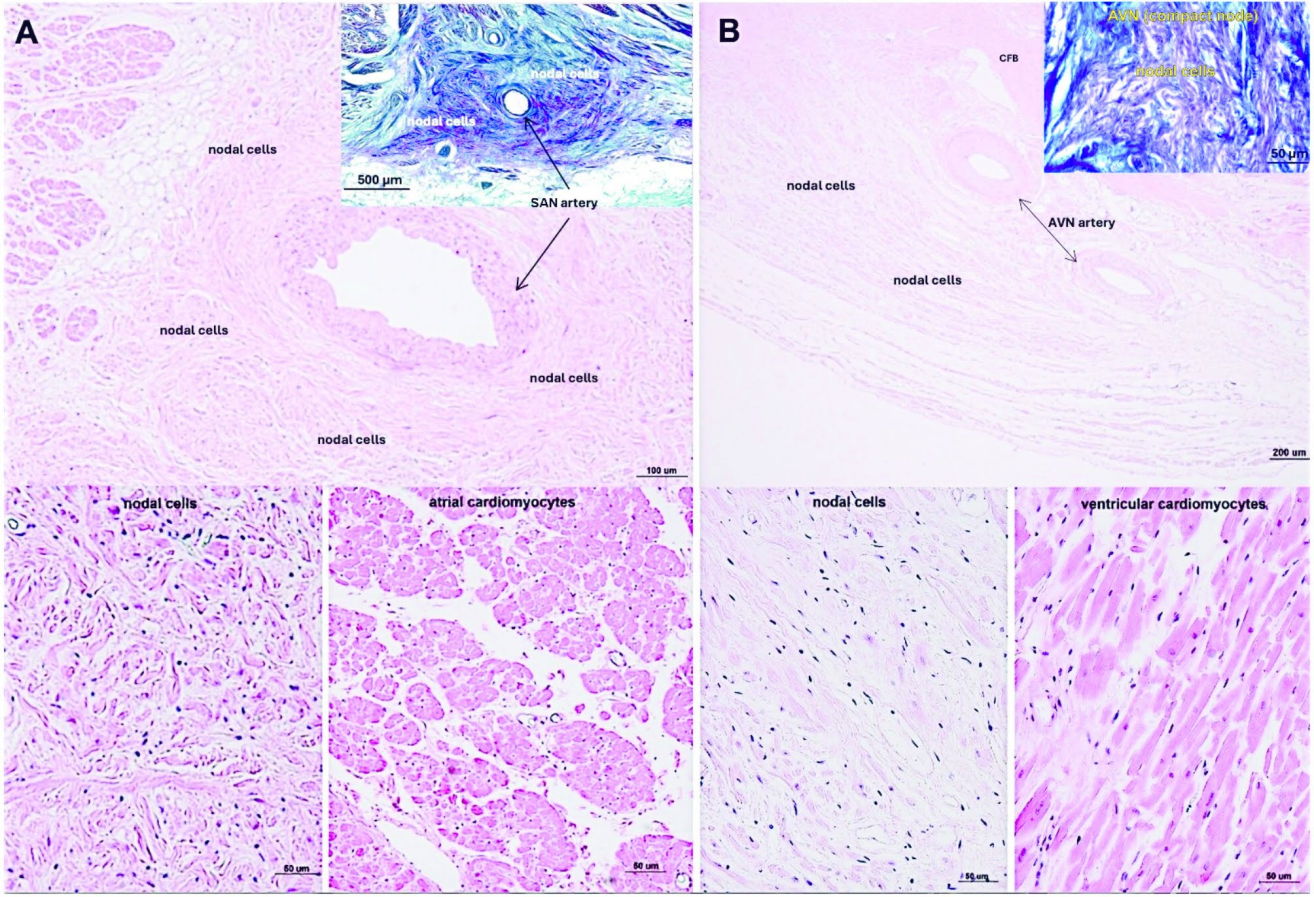
Representative images of Hematoxylin and Eosin, and Masson’s trichrome stained (**A**) sinoatrial node (SAN) and (**B**) atrioventricular node (AVN).

**Fig. 3:**
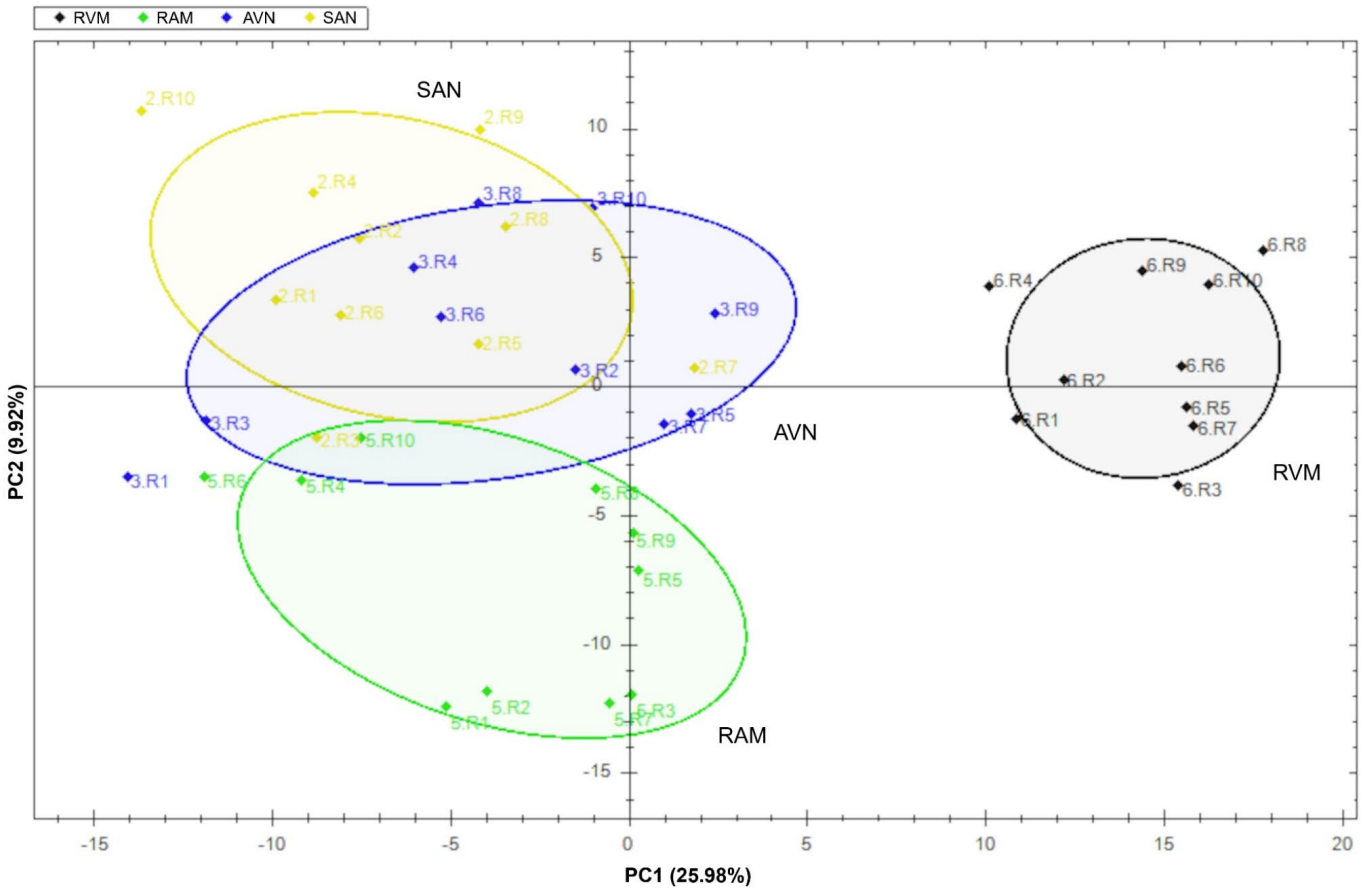
Schematic representation of the proteins identified in the data-independent acquisition proteomics experiment. Principal component analysis (PCA) of proteomics data for all analyzed hearts R1-R10 shows variation in the dataset explained by differences in protein expression between sinoatrial node (SAN), atrioventricular node (AVN), right atrial muscle (RAM) and right ventricle muscle (RVM).

**Fig. 4:**
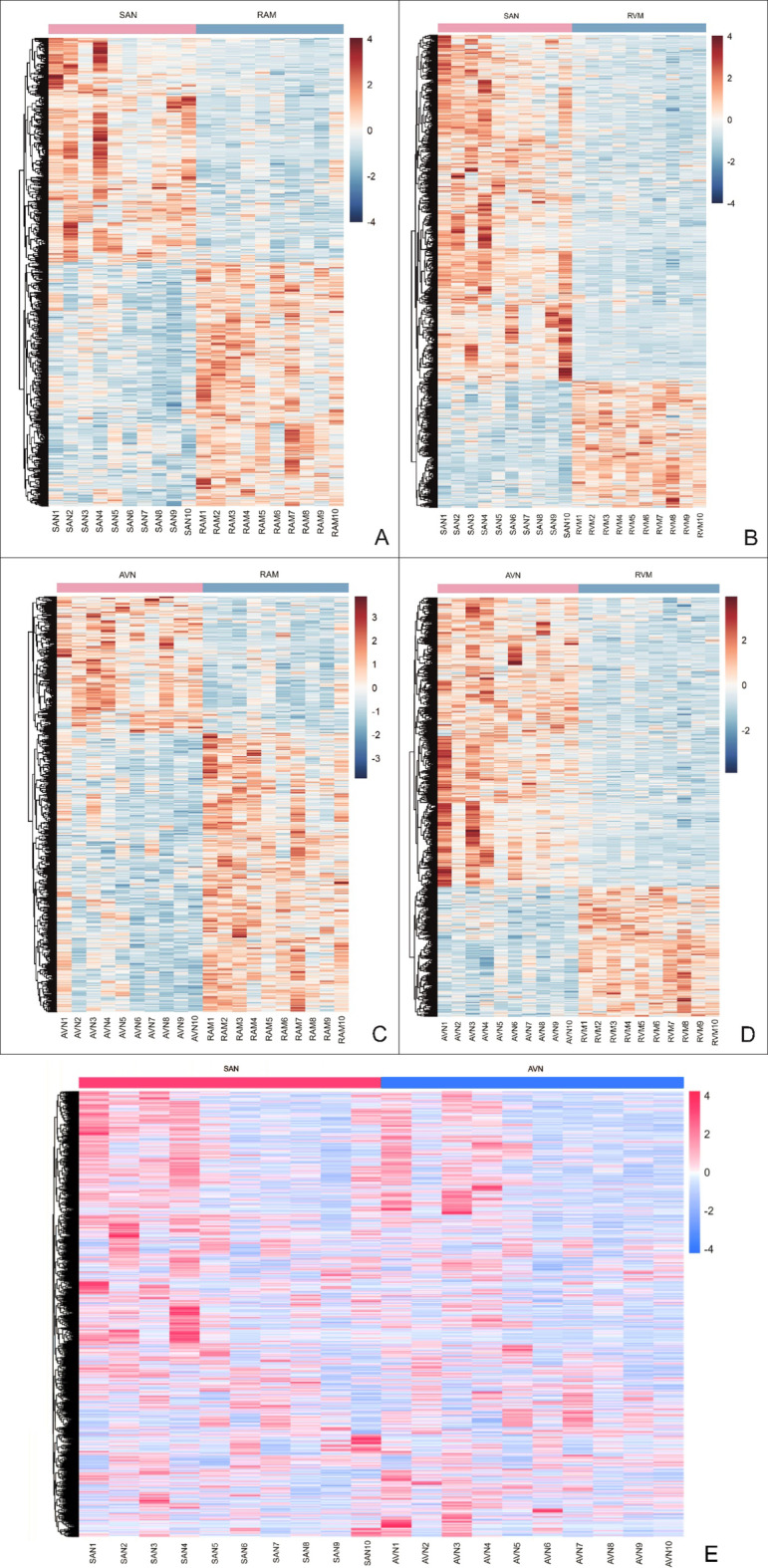
Heat maps presentation of a hierarchical cluster showing comparison of significantly changed proteins abundance for (**A**) sinoatrial node (SAN) vs. the right atrium myocardium (RAM) (**B**) SAN vs. the right ventricle myocardium (RVM) (**C**) the atrioventricular node (AVN) vs. RAM and (**D**) AVN vs. RVM and (**E**) SAN vs. AVN. On the color scale, blue and red indicate low and high protein abundance level, respectively.

Histological sections through collected SAN and AVN samples confirmed correct identification and proper dissection of cardiac conduction system nodes (Figs. 1 and 2). LC‒MS/MS measurements operated in DDA mode identified 44,152 proteotypic peptides, providing a human heart spectral library composed of 5179 protein groups. The library was used to analyze DIA runs in Spectronaut. Spectral library precursor recovery was 73.7%, and the median protein group CV was approximately 18%, which allowed for the calculation of a significant cutoff equal to a fold change of 1.25 (statistical power greater than 80%). In total, during the DIA proteomics analysis 2752 different proteins were identified in all sample sets (SAN, AVN, RAM and RVM) (Supplementary Table 1). Performed principal component analysis (PCA) allowed to show significant differences between analyzed cardiac regions (Fig. 3). The comparison between SAN and RAM revealed differential regulation in 575 proteins across these samples (359 were up-regulated in SAN, while 216 were up-regulated in the RAM). Similarly, 451 proteins showed differential regulation in AVN and RAM, with 228 upregulated in AVN and 223 upregulated in atrial working cardiomyocytes. When comparing SAN with RVM, 813 proteins were upregulated in SAN and 275 in RVM. In the comparison between AVN and RVM, 592 proteins were upregulated in AVN, while 261 were upregulated in RVM. Finally, the comparison between the two nodes revealed differential regulation of 212 proteins across the nodal samples, with 168 upregulated in the SAN and 44 upregulated in the AVN. Unsupervised hierarchical clustering (heat maps) of protein abundance resulted in grouping of SAN, AVN, RAM and RVM into distinct clusters (Fig. 4), which confirmed the good resolution quality and reproducibility of the data across samples and allowed for quantitative and qualitative analyses of differences between collected groups of cardiac tissue. To confirm the results from DIA proteomics, we performed Western blot analysis for selected, differentially regulated proteins (Supplementary Figs. 1 and 2).

### Proteins abundance analysis

Comparative analyses of tissue groups confirmed the presence of proteins distinctive for a given region of heart (tissue markers) (Supplementary Table 3). In the comparison between SAN and RVM, peptides such as: glycogen [starch] synthase, muscle (GYS1), gap junction alpha-1 protein (GJA1), ryanodine receptor 2 (RYR2), titin (TTN), troponin T, cardiac muscle (TNNT2) and cardiac phospholamban (PLN) exhibited higher abundance in working myocardium. Similarly, gap junction alpha-1 protein (GJA1), titin (TTN), troponin T, cardiac muscle (TNNT2) and cardiac phospholamban (PLN) were less abundant in AVN than in myocardial samples. Moreover, the expression of natriuretic peptides A (NPPA) was higher in the RAM than in the SAN and AVN tissue, but lower in the RVM compared to the nodal tissue (Supplementary Table 3).

Proteins constituting calcium channels such as voltage-dependent calcium channel subunit alpha-2/delta-2 (CACNA2D1) and voltage-dependent calcium channel subunit alpha-2/delta-1 (CACNA2D2) showed higher expression in the SAN and AVN in comparison to working myocardium. Moreover, vimentin (VIM) a fibroblast marker was over-represented in both nodes in comparison to RVM. Finally, collagen alpha-1(I) chain (COL1A1) showed higher expression in both nodal tissue compared to atrial and ventricular cardiomyocytes.

Mitochondrial brown fat uncoupling protein 1 (UCP1), perilipin-4 (PLIN1), fatty acid-binding protein, adipocyte (FABP4) exhibited higher abundance in SAN than AVN (Supplementary Table 3).

### Pathway enrichment analysis

**Fig. 5:**
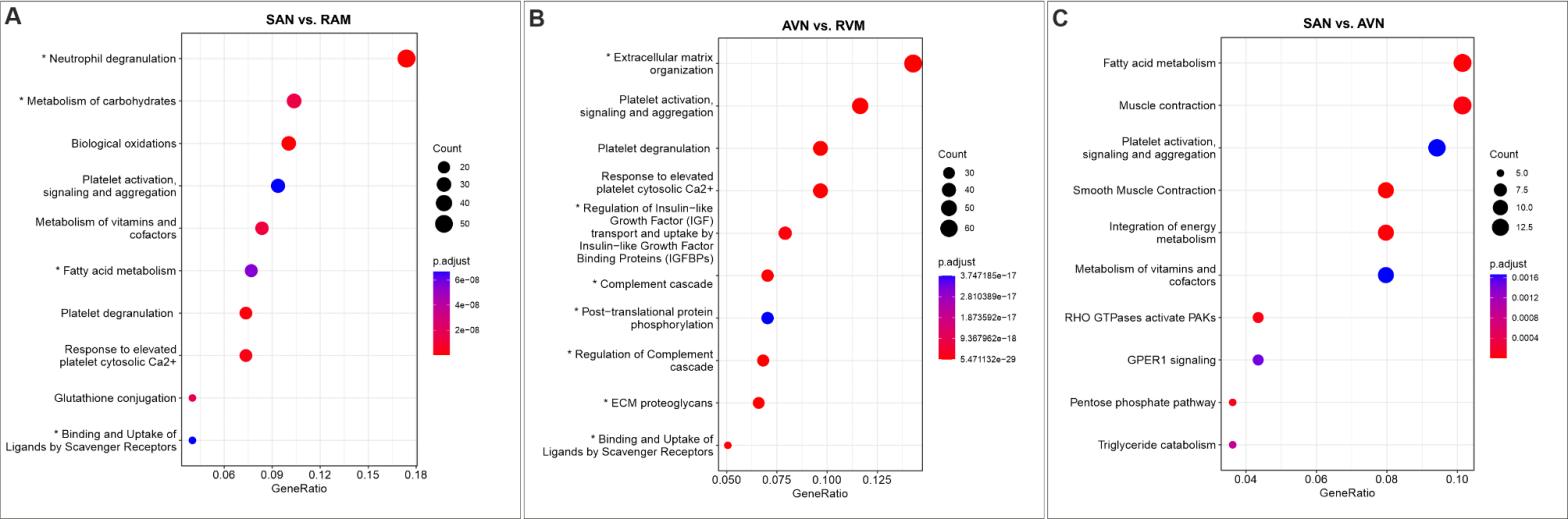
Pathway-based analyses showing (**A**) increased expression in the sinoatrial node (SAN) compared to the myocardium of right atrium (RAM), (**B**) increased expression in the atrioventricular node (AVN) compared to the myocardium of right ventricle (RVM), (**C**) increased expression in the SAN compared to the AVN. The genes encoding proteins involved in the pathways marked by * have been outlined in individual Supplementary Tables 4 and 5.

The enriched pathways were categorized based on the biological processes they are involved in. In the analysis conducted, it is noteworthy, that in comparison between SAN and RAM we found significantly enriched pathways in SAN tissue related to neutrophil degranulation, metabolism of carbohydrates, fatty acid metabolism, binding and uptake of ligands by scavenger receptors. Next, pathways such as neutrophil degranulation, extracellular matrix organization, SRP − dependent cotranslational protein targeting to membrane, complement cascade and regulation of complement cascade were upregulated in SAN tissue in comparison to RVM (Supplementary Tables 4 and 5, Fig. 5, Supplementary Fig. 3).

Similarly, in the comparative analysis of AVN with RAM, we observed that pathways related to neutrophil degranulation, extracellular matrix organization, degradation of the extracellular matrix, ECM proteoglycans, collagen chain trimerization, collagen biosynthesis and modifying enzymes were significantly more regulated in AVN than RAM (Supplementary Tables 4 and 5, Supplementary Fig. 3). Extracellular matrix organization, ECM proteoglycans, regulation of Insulin-like Growth Factor (IGF) transport and uptake by Insulin − like Growth Factor Binding Proteins (IGFBPs), complement cascade, regulation of complement cascade, post − translational protein phosphorylation and binding and uptake of ligands by scavenger receptors were more abundant in AVN than RVM (Supplementary Tables 4 and 5, Fig. 5).

On the other hand, in working myocardium pathways related to contractions, respiratory electron transport and mitochondrial translation were over-represented in comparison to nodal tissue (Supplementary Tables 6 and 7, Supplementary Fig. 4).

In comparing SAN and AVN, distinct pathway enrichments highlight the functional and structural differences between these nodal tissues (Supplementary Table 8). Pathways significantly enriched in the SAN include fatty acid metabolism, integration of energy metabolism, metabolism of vitamins and cofactors, and the pentose phosphate pathway, suggesting a predominant role for metabolic and energy processes in SAN function. Additionally, signaling pathways such as RHO GTPases activating PAKs and GPER1 signaling were overrepresented in the SAN, reflecting its specialized regulatory mechanisms (Supplementary Table 8). In contrast, the AVN showed enriched pathways associated with extracellular matrix organization, collagen biosynthesis. Notably, pathways such as integrin cell surface interactions, elastic fiber formation, and signaling by PDGF emphasize the importance of extracellular matrix organization interactions and mechanical integrity in AVN tissue (Supplementary Table 8).

### Protein interaction networks analysis

**Fig. 6:**
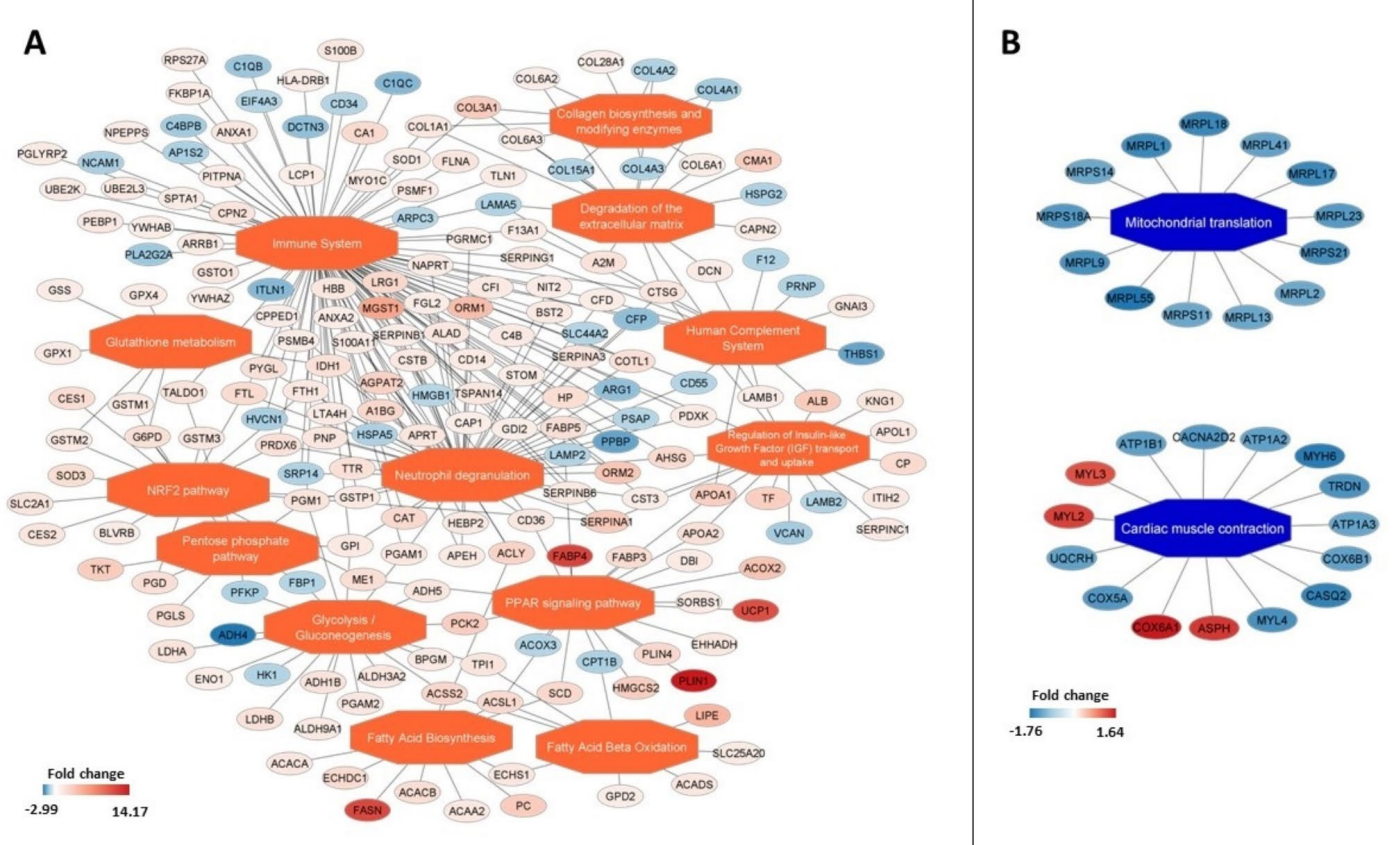
Enriched functional network upregulated (**A**) and downregulated (**B**) in sinoatrial node compared to right atrial myocardium. Activated pathways are shown as orange central nodes, and inhibited pathways are shown as blue central nodes along with red (upregulated) or blue (downregulated) protein nodes. q-value < 0.05; *n* = 10.

**Fig. 7:**
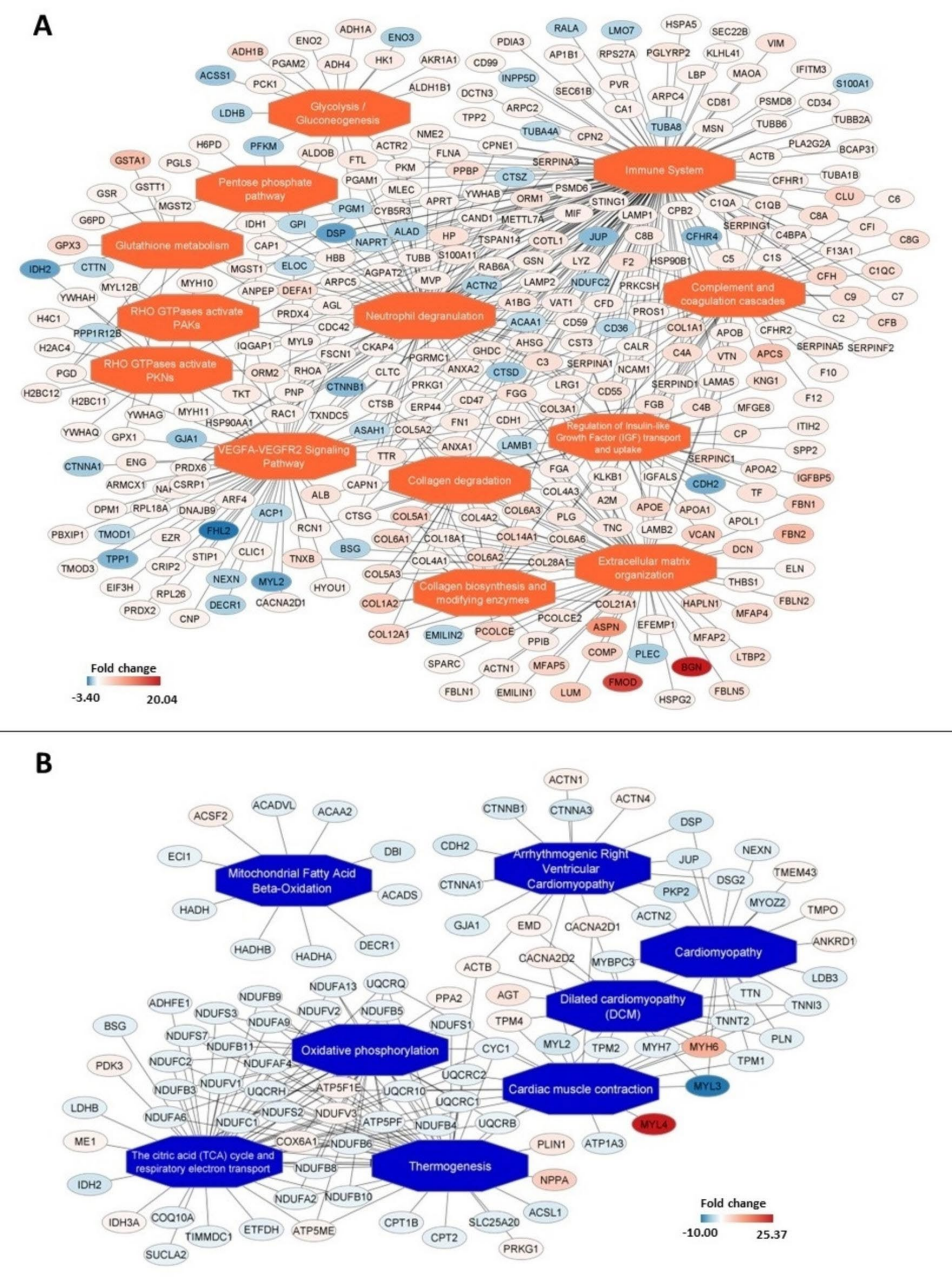
Enriched functional network upregulated (**A**) and downregulated (**B**) in atrioventricular node compared to right ventricle myocardium. Activated pathways are shown as orange central nodes, and inhibited pathways are shown as blue central nodes along with red (upregulated) or blue (downregulated) protein nodes. q-value < 0.05; *n* = 10.

**Fig. 8:**
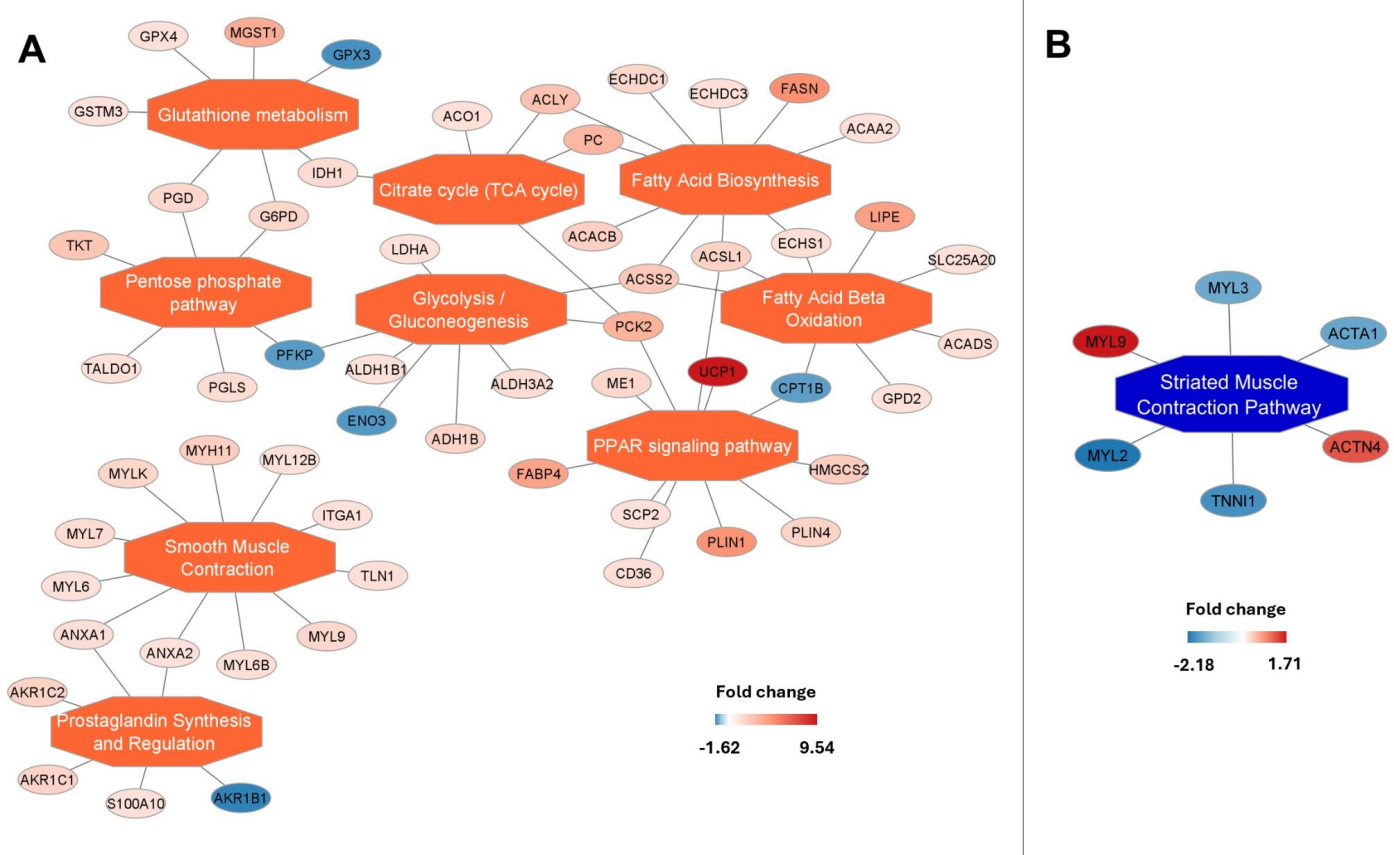
Enriched functional network upregulated (**A**) and downregulated (**B**) in sinoatrial node compared to atrioventricular node. Activated pathways are shown as orange central nodes, and inhibited pathways are shown as blue central nodes along with red (upregulated) or blue (downregulated) protein nodes. q-value < 0.05; *n* = 10.

Some common enriched pathways for nodal tissue or for working myocardium may be found among analyzed dataset. Detailed visualization of enriched pathway networks in the SAN and RAM as well as in the AVN and RVM muscle are presented in Figs. 6 and 7. Protein-coding gene assigned to common pathways for all analysis are listed in Supplementary Table 9. In both nodal tissues (compared to working myocardium), the following pathways were upregulated: regulation of IGF transport and uptake by IGFBPs, post-translational protein phosphorylation, glutathione metabolism, metabolism of carbohydrates, glycolysis and gluconeogenesis. Other common for nodal tissue pathways were these related to immune system (innate immune system, immune system, neutrophil degranulation) and related to extracellular matrix (collagen biosynthesis and modifying enzymes, collagen chain trimerization, collagen degradation, degradation of the extracellular matrix, extracellular matrix organization, collagen formation). The pathways related to cardiac muscle contraction were more abundant in the samples from RAM and RVM compared to both nodal tissues (Supplementary Table 9). Figure 8 illustrates the enriched functional networks that are differentially regulated in the SAN compared to the AVN. Notably, several key metabolic pathways are upregulated in the SAN, including the peroxisome proliferator-activated receptor (PPAR) signaling pathway, fatty acid biosynthesis and beta-oxidation, glycolysis and gluconeogenesis, glutathione metabolism, pentose phosphate pathway, the citrate cycle, as well as prostaglandin synthesis and regulatory pathways.

## Discussion

Proteome of electrical conduction cardiac system in humans has not yet been widely studied. The majority of available studies are based on the analysis of animal material^24,39,40^. One of the available study based on human tissue compared 16 anatomic cardiac regions, collected from only 3 organs, that were dissected after 72 h of donors’ death^18,20^. Another study analysed samples from human heart biopsies^41^. In both studies significant differences were found, both qualitative and quantitative, between the studied heart regions, that allows to draw a sketch of protein map for the human heart. However, this map is incomplete, as it lacks the key element – the electrical conduction system. In our previous pilot study we tried to address this gap in knowledge by analysing the proteome of the human SAN^21^. The current study is the first that presents proteomic profile of human SAN and AVN in comparison to working cardiomyocytes.

The SAN and AVN are specialized regions of the heart composed of multiple cell types, including fibroblasts, adipocytes, and immune cells, which influence their function through cell-to-cell interactions and modulation^42^. Kanemaru et al. recently investigated the human SAN microenvironment using a spatial multiomics approach, revealing a distinctive repertoire of ion channels, fibroblasts, and glial cells^43^. In our recent study, we utilized a novel machine learning deconvolution method, Bulk2space, to characterize the cellular heterogeneity of the human SAN, identifying 11 distinct cell populations, including pacemaker cardiomyocytes expressing key pacemaking ion channels (HCN1, HCN4, CACNA1D), as well as adipocyte and fibroblast populations and key immune cells^44^. Electricity in the heart is primarily driven by ion channels and their respective ionic currents. While the expression of ion channels varies significantly across different regions of the heart, a detailed discussion of this complexity is beyond the scope of this manuscript but has been reviewed extensively by Dobrzynski et al.^3^. High expression of fibroblasts within the nodal tissues^45^ provides insulation of the small, specialized nodal cardiomyocytes from the surrounding atrial myocardium and this fibrotic matrix also provides mechanical support to the nodal cells^46^. Within the nodal tissues, high expression of immune system cell^44^ such as innate macrophages have been shown to be electrically coupled to nodal cells via Cx43 and facilitate the AVN conduction^47^. Various published studies have demonstrated the complex expression of diverse cell types within the human nodes at the mRNA level, indicating that immune cells and fibroblasts do not merely passively reside in the nodes but actively contribute to their function. In our current study, we show for the first time an enriched expression of unique proteins involved in the immune system, neutrophil degranulation, collagen biosynthesis, and extracellular matrix organization within nodal tissues (Figs. 6 and 7). For instance, both nodes express proteins such as COL1A1, associated with fibroblasts, and CD34, expressed by immune cells, among other notable proteins. Notably, fibrosis linked to the COL1A1 gene has been reported to play a role in the progression of heart failure^48^. Interestingly, CD34 has been shown to be highly expressed by telocytes, a type of mesenchymal cell. In human SAN tissue, within regions where HCN4 (a nodal cell marker) is highly expressed, CD34-positive cells have been observed within the extracellular matrix. This suggests a close interaction between these cells and nodal myocytes, with a potential role in immunomodulation^49^.

The results of our study showed upregulated pathways related to extracellular matrix and immune system in the SAN, and AVN, which confirms that the nodal cells are embedded in the rich network of extracellular matrix and that immune cells influence their function. Markers for fibroblasts (COL1A1, VIM) and mast cells (TPSB2/TPSAB1) were significantly more expressed in the SAN and AVN than in RVM. Additionally, COL1A1, TPSB2/TPSAB1 and markers for fat/adipocytes cells (Fatty acid-binding protein 3, 4 and 5) were upregulated in nodes, which is consistent with previous studies^50^ Moreover, monocyte differentiation antigen CD14 was upregulated in SAN compared to RAM and RVM. The study by Jia-Hua Qu et al., which focused on diverse populations of SAN cells, suggested that the pacemaker cells exhibit suppressed metabolic activities related to fatty acids and nitrogen, along with limited immune signaling and proliferation potential. Conversely, other cell populations within the SAN, not directly responsible for heartbeat initiation, demonstrate a robust ability to proliferate and engage in immune responses. This differentiation in cellular behavior may play a crucial role in maintaining the optimal environment necessary for sustaining the healthy functioning of the SAN tissue’s pacemaker activity^51^. Cardiac macrophages may facilitate electrical conduction, and photostimulation of macrophages expressing channel rhodopsin-2 enhances atrioventricular conduction. Furthermore, the targeted removal of connexin 43 in macrophages and congenital absence of macrophages result in delayed atrioventricular conduction^47^.It is also worth noting the NRF2 pathway that contributes to the anti-inflammatory process (reactive oxygen species scavenging machinery), whose suppression have a role in development sick sinus syndrome^52^. In activation, NRF2 regulate CACNA1G, improve T-type calcium channel function, and ameliorate the SAN dysfunction^52^. Our study found that NRF2 pathway is significantly upregulated in SAN and AVN samples, which increases evidence of importance of immune cells controlling the function of the cardiac conduction system.

Another notable observation resulting from our study included upregulation of the calcium voltage-gated channel auxiliary subunit alpha2delta-1 and − 2 (CACNA2D-1 and − 2) proteins in nodal samples as the mutations identified in the CACNA2D-1 gene were previously found in Brugada syndrome patients and in individuals with an early repolarization syndrome^53^. Our study also identified gap junction alpha-1 protein (GJA1) (also known as connexin 43 - Cx43) and gap junction alpha-1 protein (GJA5) (also known as connexin 40 - Cx40) that were downregulated in SAN tissue (Supplementary Table 3). GJA1 and GJA5 are essential for rapid conduction between atrial cardiomyocytes crest^5^. Additionally, they belong to key human proteins involved in the development of the cardiac conduction system, as part of the list of 35 proteins described to play a role in its development^54,55^. Furthermore, the natriuretic peptide A (NPPA), also known as atrial natriuretic factor (ANF), showed upregulation in the SAN and AVN compared to RVM, but also downregulation when compared nodal tissue to RAM, what is consistent with our suspicions. The NPPA gene is specifically expressed in developing working myocardium, indicating the early stages of chamber formation. The regulatory mechanism for this selective expression was previously investigated development^55^ and the NPPA was explored as a protein that could potentially serve as a marker for the SAN^56^.

Available scientific reports also speculate that visinin-like protein 1 (VSNL1) may serve as a potential marker for the pacemaker cells (compared to working myocardium). In the study conducted by Dandan Liang et al. it was suspected that VSNL1 could be a marker for the SAN in the mammalian heart (mice). Researchers revealed the effect of VSNL1 in SAN through functional studies and gene expression analysis, suggesting that VSNL1 + core cells may play more significant roles in SAN function compared to other clusters^40^. However, the specific expression of VSNL1 in the SAN has not been investigated in humans so far. In our DIA proteomics analysis (validated by Western blot), we confirmed a higher abundance of VSNL1 protein in the SAN and AVN compared to the working myocardium. No differences were observed in VSNL1 protein expression between the nodes. The visinin-like protein subfamily forms a highly homologous subgroup within the neuronal calcium sensor protein family. Comparative studies indicate that VSNLs are primarily expressed in the brain, displaying restricted expression patterns in various subsets of neurons, but they are also present in peripheral organs^57^. The VSNL1 has been found to be involved in calcium signaling pathways and implicated in synaptic pathology in Alzheimer’s disease^58^. Another study has identified a series of SAN-enriched gene programs, including VSNL1, which was also found to be enriched in fetal human SAN regions^41^, suggesting its potential role in SAN development. However, its function in the SAN remains unclear.

Moreover, our study highlights several key differences between the SAN and AVN, as shown in Fig. 8. Notably, one such difference is the enrichment of the PPAR pathway in the SAN compared to the AVN. PPARs are a class of transcription factors that play a critical role in key metabolic processes, including fatty acid uptake^59^. The enriched PPAR signaling pathway in the SAN compared to the AVN suggests that this pathway plays a crucial role in glycolysis, the TCA cycle, fatty acid biosynthesis, and oxidation. This underscores the metabolic importance of the SAN as the tissue responsible for generating the heartbeat, highlighting its higher metabolic activity compared to the AVN. Furthermore, previous studies have demonstrated that PPAR activators can be used, for example, in heart failure to improve energy utilization^60^. Another study demonstrated that activation of PPAR-alpha enhances fibrosis^61^. Given the higher percentage of collagen fibers in the SAN compared to the AVN^62^, this could explain why the PPAR pathway is more prominent in the SAN. Our results also show that the pentose phosphate pathway is enhanced in the SAN compared to the AVN. This pathway, along with the TCA cycle, facilitates efficient glucose flux in and out of SAN cells, enabling the modulation of heart rate^63^. Additionally, the enhanced prostaglandin synthesis and regulatory proteins observed in the SAN may be explained by its higher content of epithelial cells, which are known to secrete prostaglandins to enhance cellular resistance to damage. This connection aligns with findings by Qu et al.^51^, who identified a significant cluster of epithelial cells, among other cell types, in the SAN.

It is now widely recognized that modern quantitative mass spectrometry techniques (especially those relying on data-independent acquisition, which was used in the present study) outperform Western blot assays for most performance characteristics (i.a. linear dynamic range, reproducibility, robustness, ability to multiplex^32,64^. This is primarily because mass spectrometry-based measurements, unlike antibody-based methods, rely on the quantitation of multiple unique peptides for each protein rather than a single epitope. This significantly enhances the selectivity, accuracy, and precision of the measurements. In this study, we further validated the expression of four selected proteins using Western blot analysis. These proteins were chosen based on their expression patterns in the SAN and AVN compared to the RVM. Notably, the expression of most of these proteins (MFAP4, GPD1, VSNL1) was markedly downregulated in the RVM relative to the SAN and AVN. These findings corroborated our shotgun proteomics data, confirming the distinct proteomic profiles of cardiac conduction system nodes.

The main limitation of the study is that the analyzed tissues are human autopsied material collected within 16 h of death, which may result in autolysis of most sensitive proteins and proteins expressed at low levels. However, autopsy is the only available source of structurally normal human heart tissue from nodal area. Although, the potential limitations associated with the use of human postmortem tissues should be taken into consideration and the data interpreted with caution, the utilization of human, not animal cardiac tissue, is greatest advantage and value of this study. Some previous studies have demonstrated that human autopsy material and explanted hearts are suitable for in-depth molecular and morphological analyses of the cardiac conduction system; therefore, the material source should not be viewed as a limitation of this project^9,10,21,28^.

Future research should prioritize the determination of SAN- and AVN-specific proteins that are upregulated in nodal areas compared to surrounding tissues, with may have potential for detailed biological identification of other components the cardiac conduction system. Further understanding of the proteome of the SAN and AVN, especially similarities and differences between nodes may explain more deeply their structure and function.

## Conclusions

The current study presents extensive comparative analysis of protein abundance in the human SAN and AVN. Few key differences may be found in the nodal proteome in comparison to working cardiomyocytes, including involvement of the immune system and upregulated pathways related to extracellular matrix. The differences between the nodes are also substantial, with the SAN showing enriched pathways such as the PPAR signaling pathway, the pentose phosphate pathway, and prostaglandin synthesis and regulatory proteins. Our results can provide the foundations for potential future protein-specific studies and experimental identification of human conduction system elements as well as implementation of proteomic strategies to identify in-depth functional differences between various heart sub-structures.

## Electronic supplementary material

Below is the link to the electronic supplementary material.

Supplementary Material 1

Supplementary Material 2

Supplementary Material 3

Supplementary Material 4

Supplementary Material 5

Supplementary Material 6

Supplementary Material 7

Supplementary Material 8

Supplementary Material 9

## Data Availability

The authors declare that the data supporting the findings of this study are available within the article and Supplementary Information files. The mass spectrometry proteomics data have been deposited to the ProteomeXchange Consortium via the PRIDE.Submission details: Project Name: Proteomics of human cardiac conduction systemProject accession: PXD051011Reviewer account details: Username: reviewer_pxd051011@ebi.ac.ukPassword: PJ4PIq1Z.
