# Supplementary figures and images for: Proteomic profile of human sinoatrial and atrioventricular nodes in comparison to working myocardium
Source: Sci Rep. 2025 Feb 28;15:7238. doi: 10.1038/s41598-025-89255-y (PMC11871314; doi:10.1038/s41598-025-89255-y)

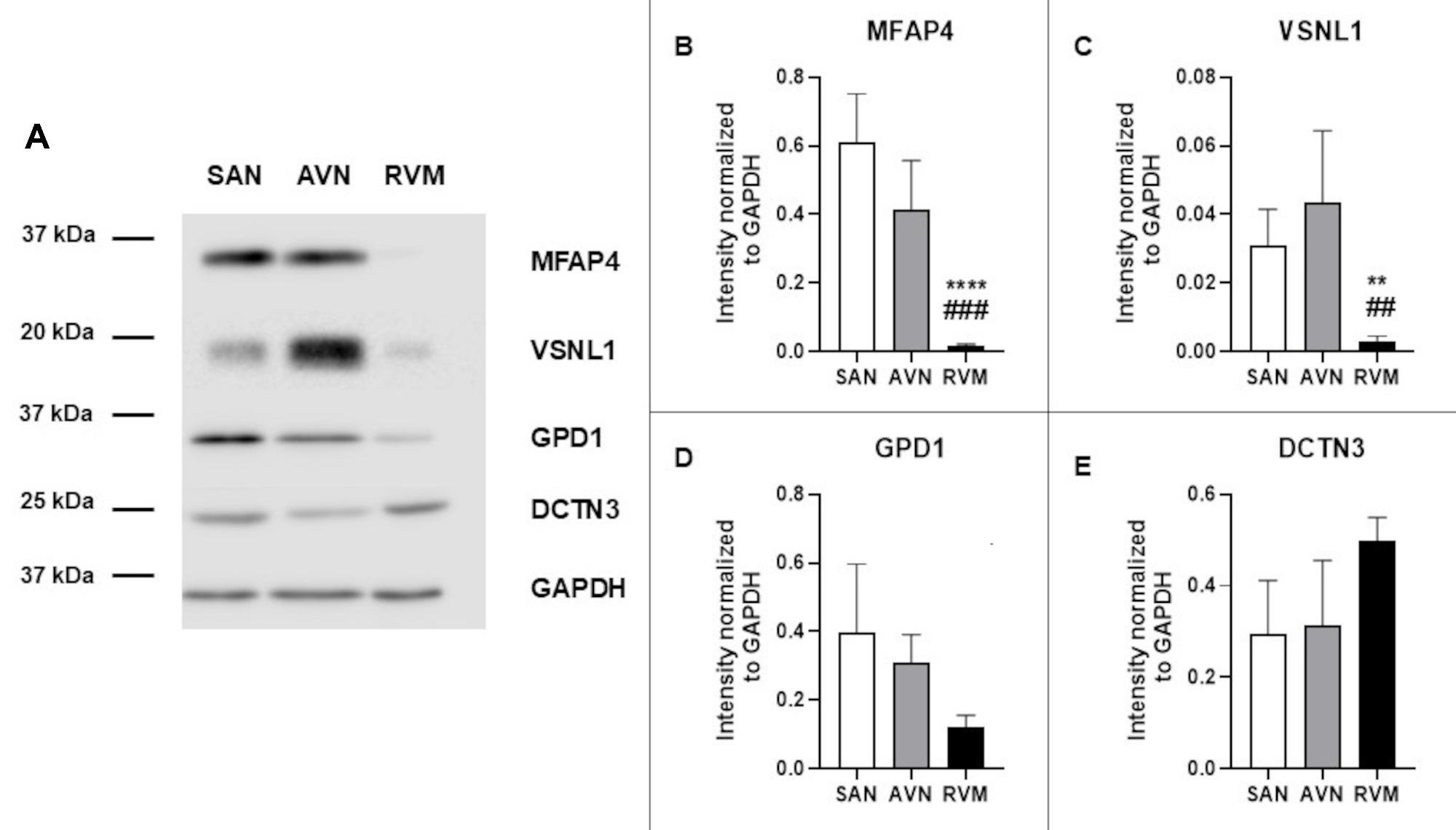

Supplement: Supplementary file 1 — Supplementary Material 1 [file 41598_2025_89255_MOESM1_ESM.jpg]

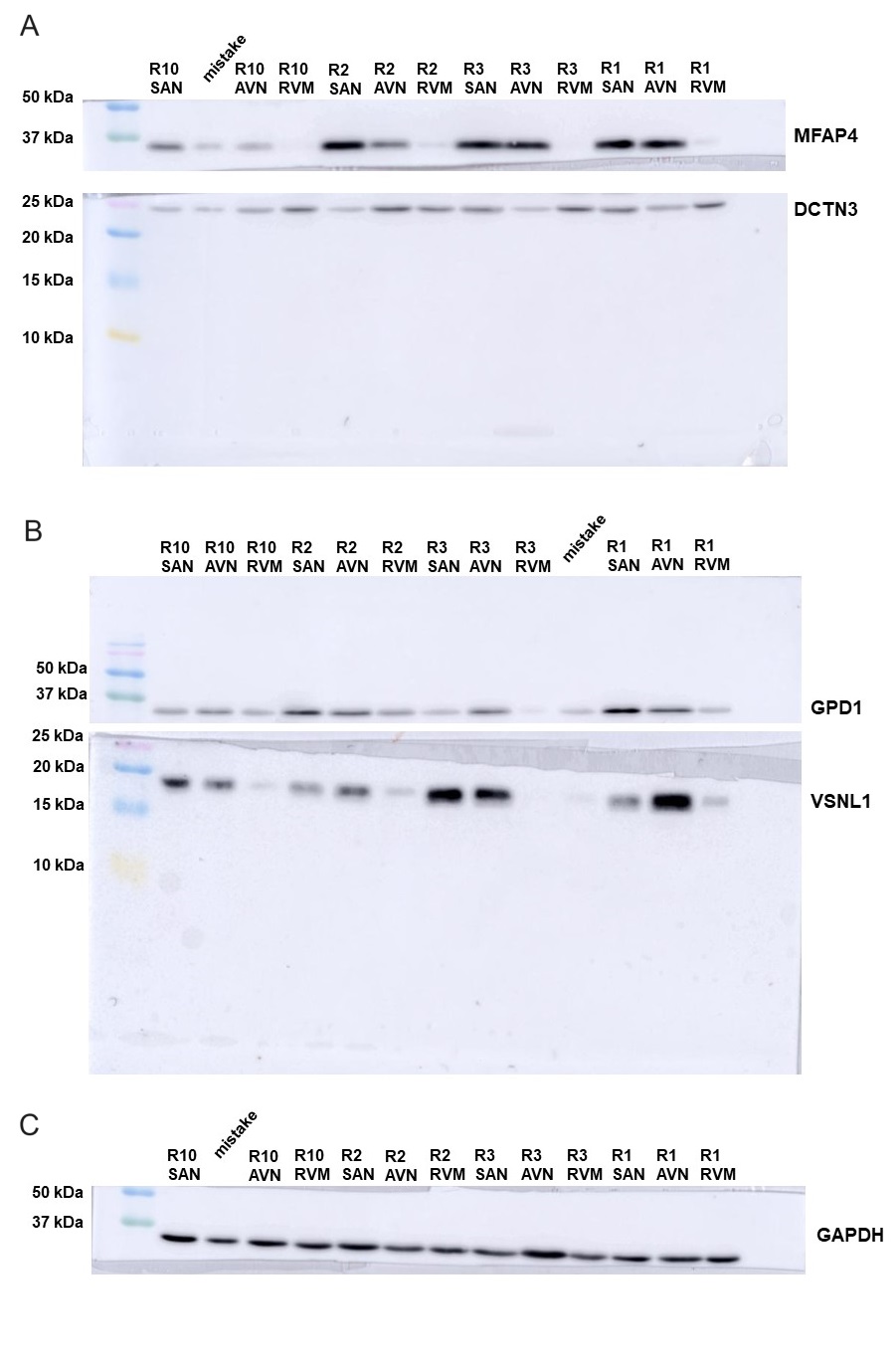

Supplement: Supplementary file 2 — Supplementary Material 2 [file 41598_2025_89255_MOESM2_ESM.jpg]

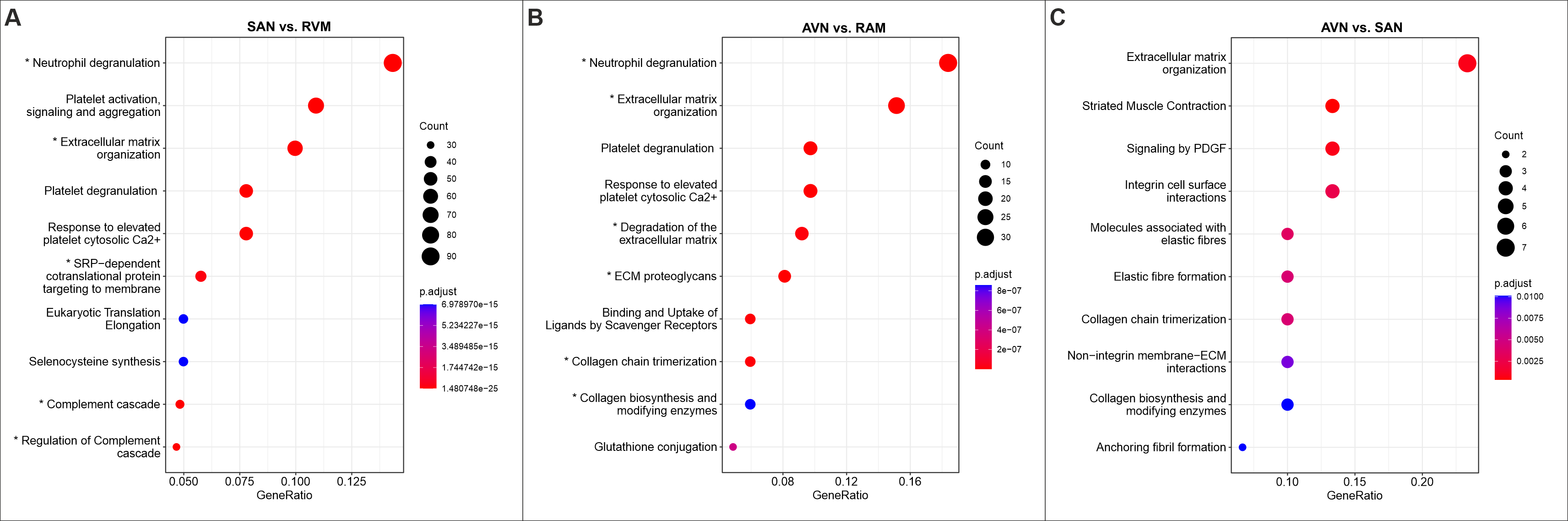

Supplement: Supplementary file 3 — Supplementary Material 3 [file 41598_2025_89255_MOESM3_ESM.png]

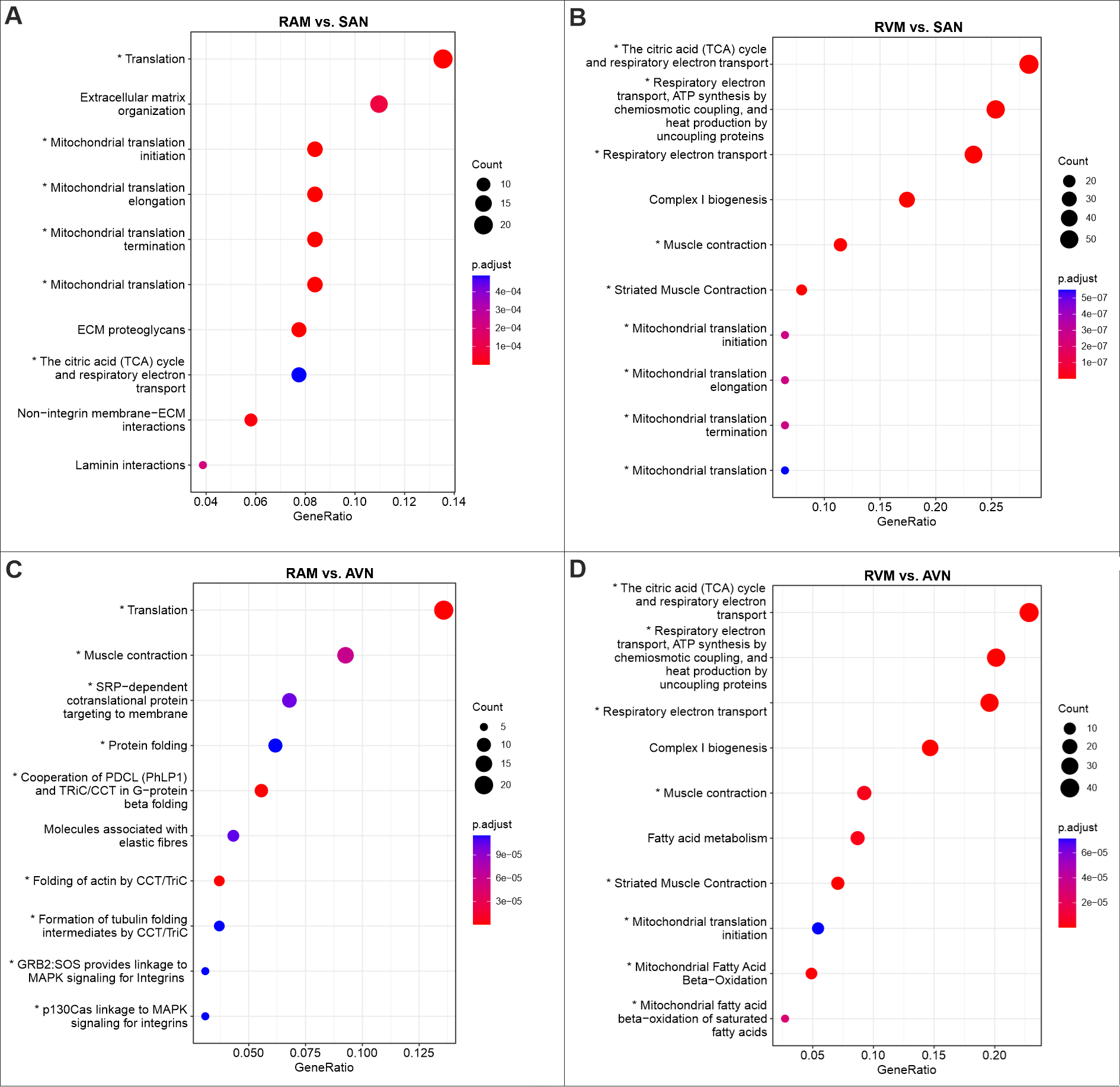

Supplement: Supplementary file 4 — Supplementary Material 4 [file 41598_2025_89255_MOESM4_ESM.png]
