# Supplementary material for: Proteomic profile of human sinoatrial and atrioventricular nodes in comparison to working myocardium
Source: Sci Rep. 2025 Feb 28;15:7238. doi: 10.1038/s41598-025-89255-y (PMC11871314; doi:10.1038/s41598-025-89255-y)
Supplement: Supplementary file 8 — Supplementary Material 8 [file 41598_2025_89255_MOESM8_ESM.docx]

Proteomic profile of human sinoatrial and atrioventricular nodes in comparison to working myocardium.

Agata Krawczyk-Ożóg, Aneta Stachowicz, Grzegorz Szoniec, Jakub Batko, Kamila Stachyra PhD, Filip Bolechała, Marcin Strona, Paweł P. Wołkow, Zeyuan Yin, Halina Dobrzynski, Mateusz K. Hołda

**Supplementary Tables 4 - 9**

**Supplementary Table 4.** Enriched pathways in sinoatrial node (SAN) in comparison to the myocardium of right atrium (RAM) and right ventricle (RVM).

| **Pathway name** | **Gene symbols of over‐represented proteins in SAN in comparison with RAM** | **Adjusted *P-value*** |
| --- | --- | --- |
| Neutrophil degranulation | MGST1, ORM1, ORM2, SERPINA1, AGPAT2, ACLY, A1BG, LRG1, CAT, AHSG, FTL, HP, FABP5, COTL1, PRDX6, PYGL, IDH1, TTR, PGM1, SERPINA3, APRT, CTSG, PNP, HBB, ALAD, TSPAN14, PGAM1, HEBP2, CSTB, CD36, FTH1, BST2, GSTP1, SERPINB6, GPI, PDXK, APEH, CD14, NAPRT, LTA4H, S100A11, SERPINB1, FGL2, PGRMC1, NIT2, CPPED1, STOM, CST3, GDI2, CFD, CAP1, ANXA2 | 6.61e-15 |
| Metabolism of carbohydrates | GALE, PC, TKT, SLC25A1, PCK2, MDH1, PGD, PGLS, G6PD, PYGL, GOT1, TALDO1, PGM1, SLC2A1, TPI1, PGAM1, PGAM2, AKR1B1, HEXA, GPI, SLC25A10, GNPDA1, BPGM, RPS27A, PGP, GNPDA2, DCN, GBE1, LUM, ALDH1A1, ENO1 | 1.40e-08 |
| Fatty acid metabolism | FASN, ACOX2, SLC25A1, ACLY, SCD, ACSL1, ACACB, ECHS1, ACADS, ACACA, ALDH3A2, EHHADH, ACAA2, PON1, GPX1, SLC25A20, LTA4H, DBI, ACSF2, GPX4, TECR, PTGR2, PTGIS | 5.50e-08 |
| Binding and uptake of ligands by scavenger receptors | COL3A1, ALB, APOA1, FTL, HPX, HP, COL1A1, HBB, HBA1, CD36, FTH1, APOL1 | 6.68e-08 |
|  | **Gene symbols of over‐represented proteins in SAN in comparison with RVM** |  |
| Neutrophil degranulation | MGST1, AGPAT2, DEFA1, COTL1, C3, HP, SERPINA3, ORM1, SERPINA1, ACLY, ORM2, A1BG, FTL, LRG1, ANXA2, AHSG, S100A11, METTL7A, CD47, VAT1, TTR, PYGL, PGRMC1, CD55, IDH1, APRT, PPBP, PRDX4, CAT, CST3, HBB, PRDX6, LYZ, GSN, CAP1, PGAM1, ACTR2, NME2, RHOA, CTSG, IQGAP1, CYB5R3, GHDC, MIF, CFD, CAND1, CD14, RAC1, TUBB, MVP, PNP, TSPAN14, TXNDC5, HSP90AA1, HVCN1, PSMD6, LAMP2, LAMP1, STING1, MLEC, RAB6A, S100A8, PDXK, CAPN1, SURF4, RHOG, STOM, RAP1B, CPNE1, PKM, ARPC5, PPIA, FGL2, ERP44, RAB7A, CD59, CCT2, CCT8, DDOST, CKAP4, CSTB, PSMD1, RAB10, FTH1, PGM2, BST1, RAB5B, XRCC6, HMGB1, SLC44A2, FABP5, GSTP1 | 1.81e-22 |
| Extracellular matrix organization | BGN, VTN, COL14A1, LUM, FBLN2, MFAP4, FBLN5, COL1A2, DCN, COL3A1, PCOLCE, FBN2, FGG, ELN, FGB, MFAP5, COL6A1, FBN1, FBLN1, PLG, COL6A2, EMILIN1, COL6A3, A2M, COL6A6, FN1, FGA, COL1A1, CD47, PPIB, COL5A3, TTR, ASPN, COL18A1, EFEMP1, TNXB, ACTN1, VCAN, TPSAB1, COL5A1, KLKB1, CTSG, COL28A1, MFAP2, LAMA4, SERPINH1, HSPG2, ITGA1, COL21A1, CMA1, CAPN1, LAMA5, NCAM1, CAPN2, VWF, COL5A2, COL4A2, FMOD, LTBP2, HTRA1, LAMB2, LTBP4, COL8A1, PCOLCE2 | 8.16e-18 |
| SRP−dependent cotranslational protein targeting to membrane | SEC61B, RPS27A, RPL26, RPL36, RPL14, RPL18, SPCS3, RPN2, RPL3, RPL18A, RPL19, RPL27A, RPS11, RPL4, SSR4, RPL7A, RPS28, RPL22, RPL28, RPS9, RPL32, SEC11A, RPS3, RPL23A, RPS25, RPS16, RPS20, RPS5, RPL23, RPL24, DDOST, RPS19, RPL12, RPL13A, RPL6, RPL15, RPS24 | 1.56e-16 |
| Complement cascade | VTN, CLU, CFH, C4A, C3, C4B, CFB, SERPING1, C8G, C9, F2, CPN2, C8A, C1QC, CFI, CFHR1, CD81, CD55, C5, C8B, C1QB, C2, C7, CFD, PROS1, C4BPA, C6, C1QA, CFHR2, C1S, CD59 | 4.43e-21 |
| Regulation of complement cascade | VTN, CLU, CFH, C4A, C3, C4B, CFB, SERPING1, C8G, C9, F2, CPN2, C8A, C1QC, CFI, CFHR1, CD81, CD55, C5, C8B, C1QB, C2, C7, PROS1, C4BPA, C6, C1QA, CFHR2, C1S, CD59 | 2.16e-23 |

Proteins were characterized by cluster and assigned to each pathway; they are listed by protein‐coding gene (Human Genome Organization gene symbol). Adjusted P‐value corresponds to the P‐value adjusted by the Bonferroni step‐down correction during the enrichment test.

**Supplementary Table 5**. Enriched pathways in atrioventricular node (AVN) in comparison to myocardium of right atrium (RAM) and right ventricle (RVM).

| **Pathway name** | **Gene symbols of over‐represented proteins in AVN in comparison with RAM** | **Adjusted *P-value*** |
| --- | --- | --- |
| Neutrophil degranulation | ORM1, ORM2, SERPINA1, TTR, A1BG, AHSG, LRG1, FABP5, HP, CAT, PRDX6, FTL, TSPAN14, MGST1, PGM1, ANPEP, CTSG, PNP, SERPINA3, CSTB, HBB, SERPINB6, BST2, FTH1, ALAD, HEBP2, GPI, GSTP1, IDH1, PGAM1, SERPINB1, LTA4H, APEH, CFD | 2.92e-10 |
| Extracellular matrix organization | FMOD, ASPN, COMP, COL12A1, CMA1, LTBP2, COL1A1, COL3A1, COL1A2, TNC, COL5A1, TTR, TNXB, TPSAB1, MFAP5, FBN2, COL28A1, FBN1, COL5A2, COL6A1, A2M, COL6A2, COL6A3, CTSG, COL5A3, MFAP2, EMILIN2, LAMB1 | 8.72e-11 |
| Degradation of the extracellular matrix | COL12A1, CMA1, COL1A1, COL3A1, COL1A2, COL5A1, TPSAB1, FBN2, FBN1, COL5A2, COL6A1, A2M, COL6A2, COL6A3, CTSG, COL5A3, LAMB1 | 1.50e-08 |
| ECM proteoglycans | FMOD, ASPN, COMP, COL1A1, COL3A1, COL1A2, TNC, COL5A1, TNXB, COL5A2, COL6A1, COL6A2, COL6A3, COL5A3, LAMB1 | 3.72e-10 |
| Collagen chain trimerization | COL12A1, COL1A1, COL3A1, COL1A2, COL5A1, COL28A1, COL5A2, COL6A1, COL6A2, COL6A3, COL5A3 | 1.03e-08 |
| Collagen biosynthesis and modifying enzymes | COL12A1, COL1A1, COL3A1, COL1A2, COL5A1, COL28A1, COL5A2, COL6A1, COL6A2, COL6A3, COL5A3 | 8.61e-07 |
| **Pathway name** | **Gene symbols of over‐represented proteins in AVN in comparison with RVM** | **Adjusted *P-value*** |
| Extracellular matrix organization | BGN, FMOD, ASPN, FBN2, COL1A2, LUM, VCAN, FBN1, PCOLCE, COL5A1, COL12A1, MFAP5, COMP, DCN, HAPLN1, COL14A1, MFAP4, COL6A1, COL1A1, COL21A1, COL6A2, FBLN2, LTBP2, TNXB, VTN, COL5A3, COL6A3, FBLN5, FGG, FGB, COL3A1, COL28A1, PLG, EMILIN1, MFAP2, PCOLCE2, TNC, COL6A6, TTR, COL4A2, A2M, COL5A2, FN1, CD47, FGA, ACTN1, PPIB, COL18A1, CDH1, EFEMP1, LAMA5, TPSAB1, FBLN1, KLKB1, NCAM1, THBS1, HSPG2, LAMB2, CTSG, CAPN1, COL4A3, COL4A1, CTSB, SPARC, ELN | 1.04e-26 |
| ECM proteoglycans | BGN, FMOD, ASPN, COL1A2, LUM, VCAN, COL5A1, COMP, DCN, HAPLN1, COL6A1, COL1A1, COL6A2, TNXB, VTN, COL5A3, COL6A3, COL3A1, TNC, COL6A6, COL4A2, COL5A2, FN1, LAMA5, NCAM1, HSPG2, LAMB2, COL4A3, COL4A1, SPARC | 5.38e-20 |
| Regulation of Insulin-like Growth Factor (IGF) transport and uptake by Insulin-like Growth Factor Binding Proteins (IGFBPs) | IGFBP5, VCAN, FBN1, APOE, C4A, SERPINC1, C3, KNG1, APOA1, C4B, ALB, F2, TF, FGG, SERPINA1, CP, PLG, TNC, APOA2, AHSG, FN1, MFGE8, ITIH2, FGA, SERPIND1, CST3, SPP2, APOB, LAMB2, CTSG, HSP90B1, PRKCSH, CKAP4, IGFALS, RCN1, APOL1 | 6.69e-19,5 |
| Complement cascade | CLU, CFH, C4A, C1QC, CFB, C3, VTN, C8A, C8G, SERPING1, C9, C4B, F2, CD55, CFI, CPN2, C1QB, CFHR1, C8B, C5, C4BPA, CD81, C2, C1QA, CFD, PROS1, C7, C6, CFHR2, CD59, C1S, CPB2 | 1.04e-26 |
| Regulation of complement cascade | CLU, CFH, C4A, C1QC, CFB, C3, VTN, C8A, C8G, SERPING1, C9, C4B, F2, CD55, CFI, CPN2, C1QB, CFHR1, C8B, C5, C4BPA, CD81, C2, C1QA, PROS1, C7, C6, CFHR2, CD59, C1S, CPB2 | 5.47e-29 |
| Post−translational protein phosphorylation | IGFBP5, VCAN, FBN1, APOE, C4A, SERPINC1, C3, KNG1, APOA1, C4B, ALB, TF, FGG, SERPINA1, CP, TNC, APOA2, AHSG, FN1, MFGE8, ITIH2, FGA, SERPIND1, CST3, SPP2, APOB, LAMB2, HSP90B1, PRKCSH, CKAP4, RCN1, APOL1 | 3.75e-17 |
| Binding and uptake of ligands by scavenger receptors | COL1A2, APOE, COL1A1, HPX, APOA1, HP, ALB, AMBP, COL3A1, FTL, COL4A2, JCHAIN, HBB, APOB, HBA1, HSP90B1, CALR, COL4A1, HSP90AA1, SPARC, APOL1, HYOU1, HPR | 3.08e-19 |

Proteins were characterized by cluster and assigned to each pathway; they are listed by protein‐coding gene (Human Genome Organization gene symbol). Adjusted P‐value corresponds to the P‐value adjusted by the Bonferroni step‐down correction during the enrichment test.

**Supplementary Table 6.** Enriched pathways in myocardium of right atrium (RAM) and right ventricle (RVM) in comparison to sinoatrial node (SAN)

| **Pathway name** | **Gene symbols of over‐represented proteins in RAM in comparison with SAN** | **Adjusted *P-value*** |
| --- | --- | --- |
| Translation | MRPL2, MRPL13, MRPS11, MRPS14, RPS29, RPS23, MRPL41, SRP14, RPS27L, RPL37A, MRPL23, MRPS18A, MRPL9, MRPS21, RPL35A, MRPL17, MRPL1, MRPL18, MRPL55, EIF3H, SSR1 | 8.00e-08 |
| Mitochondrial translation initiation | MRPL2, MRPL13, MRPS11, MRPS14, MRPL41, MRPL23, MRPS18A, MRPL9, MRPS21, MRPL17, MRPL1, MRPL18, MRPL55 | 4.67e-08 |
| Mitochondrial translation elongation | MRPL2, MRPL13, MRPS11, MRPS14, MRPL41, MRPL23, MRPS18A, MRPL9, MRPS21, MRPL17, MRPL1, MRPL18, MRPL55 | 4.67e-08 |
| Mitochondrial translation termination | MRPL2, MRPL13, MRPS11, MRPS14, MRPL41, MRPL23, MRPS18A, MRPL9, MRPS21, MRPL17, MRPL1, MRPL18, MRPL55 | 4.67e-08 |
| Mitochondrial translation | MRPL2, MRPL13, MRPS11, MRPS14, MRPL41, MRPL23, MRPS18A, MRPL9, MRPS21, MRPL17, MRPL1, MRPL18, MRPL55 | 7.29e-08 |
| The citric acid (TCA) cycle and respiratory electron transport | NDUFB9, NDUFB4, SLC16A1, PDPR, NDUFB3, ATP5F1D, COX6B1, UQCRH, ATP5PF, COX5A, NDUFS6, NDUFAF5 | 4.08e-04 |
| **Pathway name** | **Gene symbols of over‐represented proteins in RVM in comparison with SAN** | **Adjusted *P-value*** |
| The citric acid (TCA) cycle and respiratory electron transport | NDUFAF4, SDHB, ND4, NDUFS5, ETFDH, TIMMDC1, ATP5F1B, ATP5F1C, NDUFB6, NDUFAF3, COX5B, SUCLG1, ADHFE1, UQCRFS1, NDUFAB1, NDUFB10, UQCRB, COQ10A, NDUFA3, NDUFS6, CYC1, NDUFA7, NDUFA2, NDUFS8, BSG, NDUFB1, NDUFB8, UQCRQ, NDUFB2, LDHB, NDUFV2, UQCRC1, UQCRC2, NDUFS7, NDUFA10, NDUFA8, NDUFB9, ATP5F1D, NDUFB5, NDUFS3, NDUFV1, NDUFC2, NDUFS4, NDUFA6, NDUFS1, NDUFB11, NDUFA12, COX5A, NDUFS2, NDUFA13, NDUFB3, ATP5PF, NDUFA9, SLC16A1, NDUFB4, UQCRH, IDH2 | 2.90e-54 |
| Respiratory electron transport, ATP synthesis by chemiosmotic coupling, and heat production by uncoupling proteins | NDUFAF4, SDHB, ND4, NDUFS5, ETFDH, TIMMDC1, ATP5F1B, ATP5F1C, NDUFB6, NDUFAF3, COX5B, UQCRFS1, NDUFAB1, NDUFB10, UQCRB, COQ10A, NDUFA3, NDUFS6, CYC1, NDUFA7, NDUFA2, NDUFS8, NDUFB1, NDUFB8, UQCRQ, NDUFB2, NDUFV2, UQCRC1, UQCRC2, NDUFS7, NDUFA10, NDUFA8, NDUFB9, ATP5F1D, NDUFB5, NDUFS3, NDUFV1, NDUFC2, NDUFS4, NDUFA6, NDUFS1, NDUFB11, NDUFA12, COX5A, NDUFS2, NDUFA13, NDUFB3, ATP5PF, NDUFA9, NDUFB4, UQCRH | 2.90e-54 |
| Respiratory electron transport | NDUFAF4, SDHB, ND4, NDUFS5, ETFDH, TIMMDC1, NDUFB6, NDUFAF3, COX5B, UQCRFS1, NDUFAB1, NDUFB10, UQCRB, COQ10A, NDUFA3, NDUFS6, CYC1, NDUFA7, NDUFA2, NDUFS8, NDUFB1, NDUFB8, UQCRQ, NDUFB2, NDUFV2, UQCRC1, UQCRC2, NDUFS7, NDUFA10, NDUFA8, NDUFB9, NDUFB5, NDUFS3, NDUFV1, NDUFC2, NDUFS4, NDUFA6, NDUFS1, NDUFB11, NDUFA12, COX5A, NDUFS2, NDUFA13, NDUFB3, NDUFA9, NDUFB4, UQCRH | 3.33e-53 |
| Muscle contraction | ATP1B1, FGF12, MYH3, RYR2, TCAP, TNNC1, TMOD1, TTN, CAV3, TPM2, MYH8, TPM1, TNNT2, TNNI3, ATP1A3, PLN, MYBPC3, ACTN2, SORBS1, MYL1, ACTA1, MYL2, MYL3 | 2.13e-10 |
| Striated Muscle Contraction | MYH3, TCAP, TNNC1, TMOD1, TTN, TPM2, MYH8, TPM1, TNNT2, TNNI3, MYBPC3, ACTN2, MYL1, ACTA1, MYL2, MYL3 | 4.17e-17 |
| Mitochondrial translation initiation | MRPL17, MRPL15, MRPL22, MRPL47, MRPS21, MRPL2, MRPL11, MRPL41, MRPL55, MRPL18, MRPL23, MRPL1, MRPL9 | 2.66e-07 |
| Mitochondrial translation elongation | MRPL17, MRPL15, MRPL22, MRPL47, MRPS21, MRPL2, MRPL11, MRPL41, MRPL55, MRPL18, MRPL23, MRPL1, MRPL9 | 2.66e-07 |
| Mitochondrial translation termination | MRPL17, MRPL15, MRPL22, MRPL47, MRPS21, MRPL2, MRPL11, MRPL41, MRPL55, MRPL18, MRPL23, MRPL1, MRPL9 | 2.66e-07 |
| Mitochondrial translation | MRPL17, MRPL15, MRPL22, MRPL47, MRPS21, MRPL2, MRPL11, MRPL41, MRPL55, MRPL18, MRPL23, MRPL1, MRPL9 | 5.52e-07 |

Proteins were characterized by cluster and assigned to each pathway; they are listed by protein‐coding gene (Human Genome Organization gene symbol). Adjusted P‐value corresponds to the P‐value adjusted by the Bonferroni step‐down correction during the enrichment test.

**Supplementary Table 7.** Enriched pathways in myocardium of right atrium (RAM) and right ventricle (RVM) in comparison to atrioventricular node (AVN).

| **Pathway name** | **Gene symbols of over‐represented proteins in RAM in comparison with AVN** | **Adjusted  *P-value*** |
| --- | --- | --- |
| Translation | GSPT1, MRPL13, RPL11, RPN2, MRPL58, DDOST, RPS27L, RPL37A, SRP14, RPL28, RPS26, RPL36A, AIMP2, MRPS28, MRPL1, MRPL17, EIF3H, CHCHD1, MRPS18A, MRPL23, RPL35A, SSR1 | 1.97e-07 |
| Muscle contraction | ATP1A3, ATP1A2, ATP1A1, ATP1B1, MYL4, MYL6, CASQ2, MYH6, MYH11, MYL6B, FGF12, CACNA2D2, ACTG2, MYL7, NPPA | 5.96e-05 |
| SRP−dependent cotranslational protein targeting to membrane | RPL11, RPN2, DDOST, RPS27L, RPL37A, SRP14, RPL28, RPS26, RPL36A, RPL35A, SSR1 | 1.02e-04 |
| Protein folding | CCT3, CCT2, CCT4, CCT5, CCT8, ARL2, CCT6A, GNG2, GNG7, GNB4 | 1.15e-04 |
| Cooperation of PDCL (PhLP1) and TRiC/CCT in G−protein beta folding | CCT3, CCT2, CCT4, CCT5, CCT8, CCT6A, GNG2, GNG7, GNB4 | 6.76e-07 |
| Folding of actin by CCT/TriC | CCT3, CCT2, CCT4, CCT5, CCT8, CCT6A | 6.23e-07 |
| Formation of tubulin folding intermediates by CCT/TriC | CCT3, CCT2, CCT4, CCT5, CCT8, CCT6A | 1.15e-04 |
| GRB2:SOS provides linkage to MAPK signaling for Integrins | RAP1A, FN1, FGA, FGB, FGG | 1.15e-04 |
| p130Cas linkage to MAPK signaling for integrins | RAP1A, FN1, FGA, FGB, FGG | 1.15e-04 |
| **Pathway name** | **Gene symbols of over‐represented proteins in RVM in comparison with AVN** | **Adjusted  *P-value*** |
| The citric acid (TCA) cycle and respiratory electron transport | NDUFA2, NDUFB10, UQCRB, SUCLA2, NDUFB6, COQ10A, NDUFB9, ADHFE1, NDUFV2, ND4, UQCR10, CYC1, NDUFS8, UQCRC1, NDUFB5, ATP5PF, UQCRQ, NDUFB8, NDUFAF4, NDUFV1, NDUFA10, NDUFS4, NDUFS7, TIMMDC1, NDUFA12, UQCRC2, NDUFS3, NDUFC2, NDUFS1, NDUFB3, LDHB, ETFDH, NDUFS2, NDUFA6, NDUFB11, NDUFA13, NDUFA9, UQCRH, BSG, NDUFC1, NDUFB4, IDH2 | 1.15e-34 |
| Respiratory electron transport, ATP synthesis by chemiosmotic coupling, and heat production by uncoupling proteins | NDUFA2, NDUFB10, UQCRB, NDUFB6, COQ10A, NDUFB9, NDUFV2, ND4, UQCR10, CYC1, NDUFS8, UQCRC1, NDUFB5, ATP5PF, UQCRQ, NDUFB8, NDUFAF4, NDUFV1, NDUFA10, NDUFS4, NDUFS7, TIMMDC1, NDUFA12, UQCRC2, NDUFS3, NDUFC2, NDUFS1, NDUFB3, ETFDH, NDUFS2, NDUFA6, NDUFB11, NDUFA13, NDUFA9, UQCRH, NDUFC1, NDUFB4 | 3.84e-34 |
| Respiratory electron transport | NDUFA2, NDUFB10, UQCRB, NDUFB6, COQ10A, NDUFB9, NDUFV2, ND4, UQCR10, CYC1, NDUFS8, UQCRC1, NDUFB5, UQCRQ, NDUFB8, NDUFAF4, NDUFV1, NDUFA10, NDUFS4, NDUFS7, TIMMDC1, NDUFA12, UQCRC2, NDUFS3, NDUFC2, NDUFS1, NDUFB3, ETFDH, NDUFS2, NDUFA6, NDUFB11, NDUFA13, NDUFA9, UQCRH, NDUFC1, NDUFB4 | 5.66e-36 |
| Muscle contraction | TTN, TMOD1, TPM1, ACTA1, TNNT2, MYH8, TPM2, TNNI3, ACTN2, PLN, MYBPC3, ATP1A3, MYL1, FGF12, SORBS1, MYL2, MYL3 | 3.52e-06 |
| Striated Muscle Contraction | TTN, TMOD1, TPM1, ACTA1, TNNT2, MYH8, TPM2, TNNI3, ACTN2, MYBPC3, MYL1, MYL2, MYL3 | 8.74e-13 |
| Mitochondrial translation initiation | MRPS6, MRPL2, MRPL41, MRPL11, MRPL12, CHCHD1, MRPS18A, MRPL1, MRPL9, MRPL23 | 7.14e-05 |
| Mitochondrial Fatty Acid Beta−Oxidation | ECI1, HADHA, HADH, ACADS, HADHB, ACADVL, DECR1, ACAA2, DBI | 5.66e-07 |
| Mitochondrial fatty acid beta−oxidation of saturated fatty acids | HADHA, HADH, ACADS, HADHB, ACADVL | 2.74e-05 |

Proteins were characterized by cluster and assigned to each pathway; they are listed by protein‐coding gene (Human Genome Organization gene symbol). Adjusted P‐value corresponds to the P‐value adjusted by the Bonferroni step‐down correction during the enrichment test.

**Supplementary Table 8**. Enriched pathways in sinoatrial node (SAN) in comparison to atrioventricular node (AVN).

| **Pathway name** | **Gene symbols of over‐represented proteins in SAN in comparison with AVN** | **Adjusted  *P-value*** |
| --- | --- | --- |
| Fatty acid metabolism | FASN, SLC25A1, ACLY, ACACB, ACSL1, ACSF2, ALDH3A2, ACADS, TECR, ECHS1, SLC25A20, ACAA2, GPX4, SCP2 | 1.42e-05 |
| Muscle contraction | MYH11, CES1, MYLK, MYL9, MYL7, ASPH, ANXA1, FGF12, TLN1, MYL6B, MYL6, ITGA1, ANXA2, MYL12A | 4.43e-05 |
| Smooth muscle contraction | MYH11, MYLK, MYL9, MYL7, ANXA1, TLN1, MYL6B, MYL6, ITGA1, ANXA2, MYL12A | 3.70e-09 |
| Integration of energy metabolism | FASN, ACLY, GNB4, TKT, PRKAR2B, ACACB, GNG12, GNG2, CD36, TALDO1, GNAS | 2.13e-05 |
| Metabolism of vitamins and  cofactors | FASN, PC, VKORC1L1, RETSAT, CYB5A, ACACB, AKR1C1, ALDH1L1, IDH1, PDXK, MTHFD1 | 1.66e-03 |
| RHO GTPases activate PAKs | FLNA, MYH11, MYLK, MYL9, MYL6, MYL12A | 4.82e-05 |
| GPER1 signaling | GNB4, PRKAR2B, GNG12, GNG2, GNAI3, GNAS | 1.47e-03 |
| Pentose phosphate pathway | TKT, G6PD, PGD, PGLS, TALDO1 | 7.99e-05 |
| Triglyceride catabolism | PLIN1, LIPE, FABP4, MGLL, GPD2 | 8.84e-04 |
| **Pathway name** | **Gene symbols of over‐represented proteins in AVN in comparison with SAN** | **Adjusted  *P-value*** |
| Extracellular matrix  organization | EMILIN3, COL4A1, MFAP2, THBS1, COL4A2, FBN1, COL21A1 | 7.52e-04 |
| Striated muscle contraction | MYL3, ACTA1, TNNI1, MYL2 | 3.27e-04 |
| Signaling by PDGF | COL4A1, THBS1, COL4A2, THBS4 | 7.52e-04 |
| Integrin cell surface  interactions | COL4A1, THBS1, COL4A2, FBN1 | 2.56e-03 |
| Molecules associated with  elastic fibres | EMILIN3, MFAP2, FBN1 | 3.47e-03 |
| Elastic fibre formation | EMILIN3, MFAP2, FBN1 | 4.17e-03 |
| Collagen chain trimerization | COL4A1, COL4A2, COL21A1 | 4.17e-03 |
| Non−integrin membrane−ECM  interactions | COL4A1, THBS1, COL4A2 | 8.70e-03 |
| Collagen biosynthesis and  modifying enzymes | COL4A1, COL4A2, COL21A1 | 1.01e-02 |
| Anchoring fibril formation | COL4A1, COL4A2 | 1.01 e-02 |

Proteins were characterized by cluster and assigned to each pathway; they are listed by protein‐coding gene (Human Genome Organization gene symbol). Adjusted P‐value corresponds to the P‐value adjusted by the Bonferroni step‐down correction during the enrichment test.

**Supplementary Table 9**. Common enriched pathways in sinoatrial node (SAN) and atrioventricular node (AVN) in comparison to myocardium of right atrium (RAM) and right ventricle (RVM).

| **Pathway name** | **Gene symbols of over‐represented proteins in SAN and AVN in comparison with RAM and RVM** | **Adjusted *P-value***  ***SAN vs RAM***  ***AVN vs. RAM***  ***SAN vs RVM***  ***AVN vs. RVM*** |
| --- | --- | --- |
| Regulation of Insulin-like Growth Factor (IGF) transport and uptake by Insulin-like Growth Factor Binding Proteins (IGFBPs | AHSG, ALB, APOA1, APOA2, APOL1, C4B, CP, CST3, CTSG, ITIH2, KNG1, LAMB1, LAMB2, SERPINA1, SERPINC1, TF, VCAN | 4.48e-04  4.62e-14  3.15e-07  2.29e-17 |
| Post-translational protein phosphorylation | AHSG, ALB, APOA1, APOA2, APOL1, C4B, CP, CST3, ITIH2, KNG1, LAMB1, LAMB2, SERPINA1, SERPINC1, TF, VCAN | 2.91e-4  4.87e-13  1.88e-07  6.43e-16 |
| Glutathione metabolism | ANPEP, GGT5, GSTM1, GSTM2, GSTM3, GSTO1, GSTP1, GSTT1, IDH1, IDH2, MGST1, PGD | 1.47e-07  6.81e-03  7.26e-06  7.72e-04 |
| Innate Immune System | A1BG, AHSG, ALAD, ANPEP, APEH, ARPC3, ARSA, ATP6V1E1, B2M, BST2, C1QC, CAT, CCT2, CCT8, CFD, CFHR4, CFP, CSTB, CTSG, DDOST, DPP7, FABP5, FGA, FGB, FGG, FTH1, FTL, GPI, GSTP1, HBB, HEBP2, HMGB1, HP, HSP90B1, HVCN1, IDH1, ITLN1, LAMP2, LRG1, LTA4H, MAGT1, METTL7A, MGST1, ORM1, ORM2, PGAM1, PGLYRP2, PGM1, PNP, PRDX4, PRDX6, PSMF1, RAP1A, RPS27A, SERPINA1, SERPINA3, SERPINB1, SERPINB6, SERPING1, SLC44A2, SRP14, TSPAN14, TTR, UBE2K, VTN] | 2.18e-08  6.49e-21  1.29e-07  8.50e-17 |
| Immune System | A1BG, AHSG, ALAD, ANPEP, APEH, ARPC3, ARSA, ATP6V1E1, B2M, BST2, C1QC, CA1, CANX, CAT, CCT2, CCT8, CD34, CD74, CFD, CFHR4, CFP, COL1A1, COL1A2, COL3A1, CSTB, CTSG, DDOST, DPP7, EIF4A3, F13A1, FABP5, FGA, FGB, FGG, FN1, FTH1, FTL, GPI, GSTO1, GSTP1, HBB, HEBP2, HMGB1, HNRNPF, HP, HSP90B1, HSPA5, HVCN1, IDH1, ITLN1, LAMP2, LRG1, LTA4H, MAGT1, METTL7A, MGST1, NPEPPS, ORM1, ORM2, PDIA3, PEBP1, PGAM1, PGLYRP2, PGM1, PITPNA, PNP, PRDX4, PRDX6, PSMF1, RAP1A, RPS27A, SERPINA1, SERPINA3, SERPINB1, SERPINB6, SERPING1, SLC44A2, SOD1, SRP14, STAT5B, TALDO1, TSPAN14, TTR, UBE2K, UBE2L3, VTN | 1.11e-02  3.34e-07  2.73e-02  1.01e-06 |
| Neutrophil degranulation | A1BG, AHSG, ALAD, ANPEP, APEH, ARSA, B2M, BST2, CAT, CCT2, CCT8, CFD, CFP, CSTB, CTSG, DDOST, DPP7, FABP5, FTH1, FTL, GPI, GSTP1, HBB, HEBP2, HMGB1, HP, HVCN1, IDH1, LAMP2, LRG1, LTA4H, MAGT1, METTL7A, MGST1, ORM1, ORM2, PGAM1, PGM1, PNP, PRDX4, PRDX6, RAP1A, SERPINA1, SERPINA3, SERPINB1, SERPINB6, SLC44A2, SRP14, TSPAN14, TTR | 3.10e-17  2.28e-22  5.11e-14  5.08e-18 |
| Collagen biosynthesis and modifying enzymes | COL15A1, COL1A1, COL28A1, COL3A1, COL4A1, COL4A2, COL4A3, COL6A1, COL6A2, COL6A3 | 2.75e-02  3.87e-06  6.77e-06  2.97e-09 |
| Collagen chain trimerization | [COL15A1, COL1A1, COL28A1, COL3A1, COL4A1, COL4A2, COL4A3, COL6A1, COL6A2, COL6A3] | 6.42e-04  5.35e-06  3.81e-07  5.31e-10 |
| Collagen degradation | COL12A1, COL1A1, COL1A2, COL3A1, COL5A1, COL5A2, COL5A3, COL6A1, COL6A2, COL6A3, COL8A1 | ---  7.72e-04  2.78e-04  6.90e-07 |
| Degradation of the extracellular matrix | A2M, CAPN2, CMA1, COL15A1, COL1A1, COL3A1, COL4A1, COL4A2, COL4A3, COL6A1, COL6A2, COL6A3, CTSG, DCN, HSPG2, LAMA5, LAMB1 | 2.08e-04  2.58e-07  2.16e-06  1.20e-24 |
| Extracellular matrix organization | A2M, AGRN, ASPN, BGN, BMP10, CAPN2, CMA1, COL15A1, COL1A1, COL28A1, COL3A1, COL4A1, COL4A2, COL4A3, COL6A1, COL6A2, COL6A3, CTSG, DAG1, DCN, EMILIN1, EMILIN3, HAPLN1, HSPG2, LAMA4, LAMA5, LAMB1, LAMB2, LUM, MFAP5, NCAM1, THBS1, TNXB, TTR, VCAN | 9.26e-08  2.81e-18  2.69e-15 |
| Collagen formation | COL12A1, COL1A1, COL1A2, COL28A1, COL3A1, COL5A1, COL5A2, COL5A3, COL6A1, COL6A2, COL6A3, COL8A1, PPIB | ---  1.75e-04  2.34e-04  3.08e-08 |
| Metabolism of carbohydrates | AGRN, AKR1B1, FMOD, GALE, GNPDA1, GNPDA2, GOT1, GPI, HEXA, HSPG2, MDH1, PGAM1, PGAM2, PGD, PGLS, PGM1, PGP, RPS27A, SLC2A1, SORD, TALDO1, TKT, TPI1 | 6.47e-12  1.08e-06  1.48 e-03  1.20e-04 |
| Glycolysis and gluconeogenesis | GOT1, GPI, LDHA, LDHB, MDH1, PGAM1, PGAM2, SLC2A1, TPI1 | 6.32e-08  7.75e-06  7.13e-04  9.40e-05 |
|  | **Gene symbols of over‐represented proteins in RAM, RVM in comparison with SAN and AVN** |  |
| Cardiac muscle contraction | ATP1A1, ATP1A2, ATP1A3, ATP1B1, CACNA2D1, CACNA2D2, CASQ2, MYH6, MYL2, MYL3, MYL4 | 7.43e-05  4.65e-11  5.01e-03  5.14e-07 |

Proteins were characterized by cluster and assigned to each pathway; they are listed by protein‐coding gene (Human Genome Organization gene symbol). Adjusted P‐value corresponds to the P‐value adjusted by the Bonferroni step‐down correction during the enrichment test.
