# Supplementary material for: Proteomic profile of human sinoatrial and atrioventricular nodes in comparison to working myocardium
Source: Sci Rep. 2025 Feb 28;15:7238. doi: 10.1038/s41598-025-89255-y (PMC11871314; doi:10.1038/s41598-025-89255-y)
Supplement: Supplementary file 9 — Supplementary Material 9 [file 41598_2025_89255_MOESM9_ESM.docx]

**Supplementary Figure 1** Representative Western blot showing comparative expression of analyzed proteins in sinoatrial node (SAN), atrioventricular node (AVN) and right ventricle myocardium (RVM) samples (A). MFAP4 - microfibril-associated glycoprotein 4 antibody (1: 1000, Proteintech), VSNL1 - visinin-like protein 1 antibody (1:5000, Proteintech), GPD1 - glycerol-3-phospate dehydrogenase antibody (1:1000, Proteintech); DCTN3 - dynactin subunit 3 antibody (1:1000, BT LAB), GAPDH - glyceraldehyde 3-phosphate dehydrogenase (1:2000, MyBioSource). Quantitative analysis of Western blots for MFAP4 (B) , VSNL1 (C), GPD1 (D) and DCTN3 (E) expression in SAN, AVN and RVM. Mean ± SEM; **p<0.01, ****p<0.0001 compared to SAN; ^##^p<0.01, ^###^p<0.001 compared to AVN; n=4.

**Supplementary Figure 2 A, B, C.** Representative uncropped Western blot showing comparative expression of analyzed proteins in sinoatrial node (SAN), atrioventricular node (AVN) and right ventricle myocardium (RVM) samples. MFAP4 - microfibril-associated glycoprotein 4 antibody (1: 1000, Proteintech), VSNL1 - visinin-like protein 1 antibody (1:5000, Proteintech), GPD1 - glycerol-3-phospate dehydrogenase antibody (1:1000, Proteintech); DCTN3 - dynactin subunit 3 antibody (1:1000, BT LAB), GAPDH - glyceraldehyde 3-phosphate dehydrogenase (1:2000, MyBioSource).

**Supplementary Figure 3** Pathway-based analyses showing (A) increased expression in the sinoatrial node (SAN) compared to the right ventricle myocardium (RVM), (B) increased expression in the atrioventricular node (AVN) compared to the right atrial myocardium (RAM) and (C) increased expression in the AVN compared to the SAN. The genes encoding proteins involved in the pathways marked by * have been outlined in individual Supplementary Tables 4 and 5.

**Supplementary Figure 4** Pathway-based analyses showing (A) increased expression in the myocardium of right atrium (RAM) compared to the sinoatrial node (SAN), (B) increased expression in the right ventricle myocardium (RVM) compared to SAN (C) increased expression in the RAM compared to the atrioventricular node (AVN) and (D) increased expression in the RVM compared to the AVN. The genes encoding proteins involved in the pathways marked by * have been outlined in individual Supplementary Tables 6 and 7.

**Supplementary Table 1**. Protein quantities for all identified and quantified proteins in sinoatrial node (SAN), atrioventricular node (AVN), right atrial muscle (RAM) and right ventricle muscle (RVM). R1-R10 – number of analyzed heart

**Supplementary Table 2.** The list of identified peptides and their quantities for all identified and quantified proteins in sinoatrial node (SAN), atrioventricular node (AVN), right atrial muscle (RAM) and right ventricle muscle (RVM). R1-R10 – number of analyzed heart

**Supplementary Table 3.** Differentially expressed proteins in sinoatrial node (SAN), atrioventricular node (AVN), right atrial muscle (RAM) and right ventricle muscle (RVM) (fold change (FC) ≥1.25 or FC ≤ -1.25; q<0.05; n=10).

**Supplementary Tables 4 – 9 (see file).**
